# Nurse-Delivered Telehealth in Home-Based Palliative Care: Integrative Systematic Review

**DOI:** 10.2196/73024

**Published:** 2025-05-05

**Authors:** Cong Ma, Yifan Fang, Hui Zhang, Ying Zheng, Ying Zhang, Wanchen Zhao, Ge Yan, Yaoxin Zeng, Yanwu Zhang, Xiaohong Ning, Zhimeng Jia, Na Guo

**Affiliations:** 1 School of Nursing Chinese Academy of Medical Sciences & Peking Union Medical College Beijing China; 2 Department of Nursing Peking Union Medical College Hospital Chinese Academy of Medical Sciences & Peking Union Medical College Beijing China; 3 Palliative Medicine Center Peking Union Medical College Hospital Chinese Academy of Medical Sciences & Peking Union Medical College Beijing China; 4 Institute of Medical Information (IMI) & Medical Library Chinese Academy of Medical Sciences & Peking Union Medical College Beijing China; 5 School of Population Medicine and Public Health Chinese Academy of Medical Sciences & Peking Union Medical College Beijing China; 6 Temmy Latner Centre for Palliative Care Mount Sinai Hospital Toronto, ON Canada; 7 Department of Family and Community Medicine University of Toronto Toronto, ON Canada

**Keywords:** nurse, homecare services, palliative care, systematic review, telehealth, telemedicine, technology, implementation science

## Abstract

**Background:**

Telehealth technologies can enhance patients’ and their families’ access to high-quality resources in home-based palliative care. Nurses are deeply involved in delivering telehealth in home-based palliative care. However, no previous integrative systematic reviews have synthesized evidence on nurses’ roles, facilitators, and barriers to implementing nurse-delivered telehealth in home-based palliative care.

**Objective:**

This integrative systematic review aimed to provide a comprehensive understanding of the roles of nurses and the multilevel facilitators and barriers to implementing nurse-delivered telehealth in home-based palliative care, which could inform future policy development, research, and clinical practice.

**Methods:**

This integrative systematic review was conducted using Joanna Briggs Institute methodological guidance. We followed the PRISMA (Preferred Reporting Items for Systematic Reviews and Meta-Analysis) guidelines. We systematically searched articles published from January 1, 2014, to May 2024 in PubMed, Embase, Web of Science, CINAHL, and Cochrane Library. We included English-language; peer-reviewed; original; and qualitative, quantitative, and mixed methods studies that centered on nurse-delivered telehealth in home-based palliative care. We used the Mixed Methods Appraisal Tool to assess the quality of the included articles. Furthermore, 3 authors independently assessed eligibility, extracted data, and assessed the quality of articles. The entities to extract were identified by research questions of interest regardless of the type of study. We applied a convergent synthesis approach to integrate quantitative and qualitative data. Guided by the updated Consolidated Framework for Implementation Research (CFIR) 2.0, we synthesized the facilitators and barriers to implementing nurse-delivered telehealth in home-based palliative care.

**Results:**

This integrative systematic review identified 4819 unique articles, including 34 papers encompassing 29 unique primary research studies. Innovations were mainly delivered by nurses (n=8) and nurse-involved multiprofessional teams (n=18). The roles of nurses in telehealth home-based palliative care involve palliative care nurses, community nurses, nurse coordinators, nurse coaches or nurse navigators, and nurse case managers. Guided by CFIR 2.0, facilitators and barriers to implementing nurse-delivered, telehealth, home-based palliative care were identified to 6 implementation levels and 20 constructs. The key facilitators included the COVID-19 pandemic, cost avoidance to the health care system, engagement of patients and their family caregivers, and so on. The barriers included a lack of reimbursement and payment mechanisms, technical problems, insufficiently trained health care providers, and so on.

**Conclusions:**

This integrative systematic review synthesizes evidence on nurses’ evolving roles in telehealth home-based palliative care and identifies multilevel facilitators and barriers to nurse-delivered, home-based palliative care implementation. With the empowerment of telehealth technologies, nurses could establish a stronger professional identity and develop leadership in home-based palliative care. Nurses should leverage influence to promote nursing practice, clinical management, and policy support in the implementation of telehealth home-based palliative care.

**Trial Registration:**

PROSPERO CRD42024541038; https://www.crd.york.ac.uk/PROSPERO/view/CRD42024541038

## Introduction

Many patients in palliative care and their families prefer to have the patient receive care and pass away at home [1]. To satisfy patients’ preference to stay at home, home-based palliative care service has been developed, which has been shown to increase the likelihood of dying at home [2]. Home-based palliative care is a form of palliative care provided by informal caregivers (such as family members) and a trained multiprofessional team of doctors, nurses, social workers, and others in patients’ homes [3]. Compared with inpatient or acute palliative care services, home-based palliative care is more appropriate for patients with low to moderate symptom burdens and provides continuity of care for individuals who are homebound [4]. When patients experience an exacerbation of their health condition, they are often admitted to hospice or hospital for care, which is against patients’ preference for dying at home and underscores the urgent need to deliver high-quality, home-based palliative care [5]. However, the further development of home-based palliative care faces challenges, including the uneven distribution of medical resources, a shortage of specialized human resources in palliative care, and inadequate preparedness of home-based palliative care among family caregivers [6]. The development of telehealth technology offers a transformative solution to these barriers, which shows considerable potential to enhance and expand these services in home-based palliative care.

According to the US Department of Health and Human Services, telehealth allows health care providers (such as nurses, physicians, etc) to provide health services for patients and their caregivers when they are not in the same location [7]. The implementation of telehealth had extremely rapid development during the COVID-19 pandemic. In 2020, the Centers for Medicare and Medicaid Services in the United States approved 7 types of telehealth (such as live video, remote patient monitoring, audio-only visits, and case-based teleconferencing) [8,9]. Studies suggest that nurse-delivered telehealth can significantly enhance access to high-quality palliative care resources for patients, empower family caregivers, and reduce unscheduled hospitalization in the final life of patients [10].

As the primary providers of home-based palliative care, nurses play a unique role in implementing telehealth technologies into patients’ symptom management, emotional support, remote monitoring, health education, and transitional care [8,9,11,12]. Previous literature reviews regarding the use of telehealth technologies for home-based palliative care have primarily concentrated on the use of video consultation [13]; the interventions for family caregivers [8]; as well as the experiences and perspectives of patients, informal caregivers, and health care providers [14-17]. While there is a scoping review summarizing 3 main types of nurse-led palliative care models in resource-limited regions, it has not systematically explored the contextual factors to implement nurse-delivered, telehealth, home-based palliative care [18]. In conclusion, the roles of nurses in delivering telehealth home-based palliative care and the facilitators and barriers of nurse-delivered, telehealth, home-based palliative care remain unclear. Therefore, we aim to further our understanding of nurse-delivered, telehealth, home-based palliative care in this integrative systematic review. To the best of our knowledge, this is the first review of existing evidence on the roles of nurses and the influence factors in nurse-delivered, telehealth, home-based palliative care implementation.

Nurse-delivered care mainly included two approaches: (1) nurses deliver care as members of a multiprofessional team, and (2) nurses take responsibility for the leadership roles, beyond care delivery, with support from a multiprofessional team as needed [19]. Nurse-delivered, telehealth, home-based palliative care involves multiple components, including who delivers (nurses, as leaders or members of a multiprofessional team) the intervention (home-based palliative care), where (patients are at home settings, nurses are from any settings), to whom (patients and their family caregivers), and how (telehealth technologies, such as video consultation and telemonitoring; adapted from Brereton et al [20]). We use “innovation” to refer to the nurse-delivered, telehealth, home-based palliative care interventions and implementations [21].

Our research questions are as follows: (1) what are the roles of nurses in telehealth home-based palliative care? and (2) what are the facilitators and barriers to implementing nurse-delivered, telehealth, home-based palliative care? By synthesizing existing evidence and experiences on nurse-delivered, telehealth, home-based palliative care, this integrative systematic review aims to provide a comprehensive understanding of the roles of nurses and the multilevel facilitators and barriers to the implementation of nurse-delivered, telehealth, home-based palliative care, which could inform future policy development, research, and clinical practice.

## Methods

### Design

We conducted this integrative systematic review using Joanna Briggs Institute (JBI) methodological guidance [22] and following PRISMA (Preferred Reporting Items for Systematic Reviews and Meta-Analysis) guidelines [23] (Multimedia Appendix 1). This type of systematic review allows quantitative and qualitative data resources to be extracted and synthesized [24]. The term integrative systematic review is often used interchangeably with mixed studies review [25]. Integrative systematic reviews have the potential to contribute to nursing theory development, informing research, practice, and policy initiatives [24].

### Search Strategy

We developed a detailed search strategy informed by previous literature [8,13,26,27] and refined by a research librarian (Y Zhang) (Multimedia Appendix 2). There are three main parts in the search strategy: (1) terms related to “palliative care,” including the clinical content of palliative care, such as symptom management, spiritual support, grief support, etc; (2) terms related to “telehealth”; and (3) terms related to “nursing.” The terms in each part are linked with “OR,” and the terms among the 3 parts are linked with “AND.” We included literature from PubMed, Embase, Web of Science, CINAHL, and Cochrane Library from January 1, 2014, to May 2024. We also manually searched reference lists of included studies.

### Inclusion and Exclusion

Inclusion and exclusion criteria were formulated following the SPIDER (Sample, Phenomenon of Interest, Design, Evaluation, and Research type) framework, as detailed in Table 1 [28]. English-language; peer-reviewed; and primarily qualitative, quantitative, and mixed methods studies that focused on nurse-delivered telehealth in home-based palliative care were included. Given the rapid advancements in telehealth technologies, studies conducted too early may have limited relevance to technological contemporaneity and clinical applicability of findings. Therefore, we restricted our literature search to studies published within the past decade (from January 1, 2014, to May 2024).

**Table 1:** Eligibility criteria (based on the SPIDER^a^ framework).

SPIDER framework	Inclusion	Exclusion
Sample	Adult patients (aged 18 years and older) receiving palliative care, their families, caregivers, and health care providers (mainly nurses) were included	Patients with active suicidal ideation
Phenomenon of interest	Telehealth home-based palliative care delivered by nurses was included	Telehealth technologies are only used to collect data
Design	Qualitative, quantitative, and mixed methods research	Protocol proposals
Evaluation	Quality of life, symptom burden, depression, anxiety, medical resource use, satisfaction, feasibility, acceptability, experiences, attitudes, views, etc	Not applicable
Research type	Empirical studies	Not applicable

^a^SPIDER: Sample, Phenomenon of Interest, Design, Evaluation, and Research type.

The target population included adult patients (aged 18 years and older) receiving palliative care, as well as their families, caregivers, and health care providers (mainly nurses), according to the International Association for Hospice and Palliative Care [29]. The phenomenon of interest included telehealth home-based palliative care delivered by nurses.

Patients were excluded if they had an active suicidal ideation. In addition, protocol proposals were excluded because it is difficult to assess influence factors before implementation in the real world. Telehealth technologies only used to collect data were excluded because the application of telehealth technologies did not refer to intervention or implementation.

### Study Selection

The titles and abstracts were screened in the first round, and the full texts were screened in the second round. Each round was screened by 2 researchers (CM and HZ) independently. Any discrepancies were resolved by consensus among the 2 researchers and the senior researchers (Y Zheng and Y Zeng). EndNote 21.2 (Clarivate) and the web-based program “Covidence” were used to manage references. Covidence facilitated references screening.

### Quality Assessment

The methodological quality of the studies was assessed independently by 3 researchers (CM, YF, and WZ) using the Mixed Methods Appraisal Tool (MMAT; version 2018) [30]. Any discrepancies were resolved by consensus between the 2 researchers and the other senior researcher (Y Zeng). The MMAT is a critical appraisal tool designed for the quality assessment of systematic mixed studies reviews and integrative systematic reviews, which uses 5 criteria to score each study [30]. The development team of the MMAT did not advise calculating an overall score from the ratings of each criterion or excluding studies based solely on low methodological quality [30].

### Data Extraction

The entities to extract were identified by research questions of interest regardless of the type of study, which included the characteristics of articles (author, publication year, country, program name, aim, main findings, and quality assessment) and the study design of primary research (methodology, setting, sample, outcome, and measurement). We also extracted the innovations’ elements (why, what, who delivered, to whom, how, where, when, how much, and how well) according to the TIDieR (Template for Intervention Description and Replication) checklist [31]. Furthermore, 2 researchers extracted the entities and a third researcher checked the accuracy of the data (MC, FYF, and ZWC). Researchers contacted the corresponding authors to obtain information that was not available in the literature by email or ResearchGate. Data were captured across multiple Microsoft Excel spreadsheets. The roles of nurses in delivering telehealth home-based palliative care are synthesized according to extracted data.

### Data Transformation, Integration, and Synthesis

Guided by JBI methodological guidance, we applied a convergent synthesis approach to integrate quantitative and qualitative data, which involves transforming data into a mutually compatible format [22]. We also applied qualitizing, one of the data transformation methods to convert quantitative data into themes, categories, typologies, or narratives [22]. Qualitizing involves extracting data from quantitative studies and translating or converting it into textual descriptions to allow integration with qualitative data [22]. The transformed quantitative data and qualitative data are assembled simultaneously.

**Figure 1:**
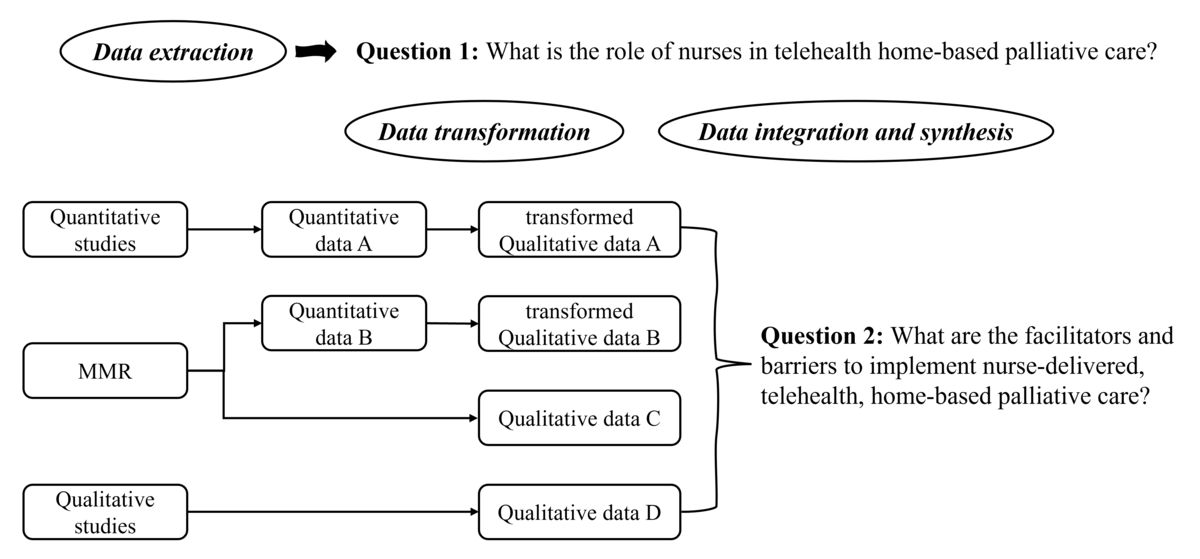
Flow diagram of data extraction, transformation, integration, and synthesis. MMR: mixed methods research.

First, the quantitative data from both the quantitative study and the mixed methods research were transformed into qualitative data in the form of a narrative summary [22]. Second, the qualitative data (ie, results section) from the qualitative study and the mixed methods research, along with the transformed quantitative data, were imported into NVivo 14 (Lumivero). Third, guided by the updated Consolidated Framework for Implementation Research (CFIR) 2.0, the facilitators and barriers to implementing nurse-delivered, telehealth, home-based palliative care were synthesized. The flow diagram is detailed in Figure 1. Adopting a hybrid deductive-inductive approach, we synthesized qualitative data at three phrases: (1) initially, we used the themes identified by the authors to code the primary qualitative data; (2) then, we extracted secondary themes under the corresponding constructs of CFIR 2.0; and (3) finally, we categorized these secondary themes into different levels. The results of qualitative studies included 3 elements—author-defined themes, author’s description of themes, and primary data [32].

The CFIR is one of the most commonly used determinant frameworks to assess contextual factors that influence implementation in the real world [21], which is appropriate for exploring the interesting questions of this review. CFIR 2.0 contains 48 constructs and 19 subconstructs across 5 domains (innovation domain, outer setting domain, inner setting domain, individuals domain, and implementation process domain), with the individuals domain including 2 subdomains (roles subdomain and characteristics subdomain) [21]. The characteristics subdomain is based on the capability, opportunity, motivation, behavior system or role-specific theories [21], which could document the characteristics applicable to the roles of nurses in telehealth home-based palliative care.

## Results

### Overall Characteristics of the Included Studies

**Figure 2:**
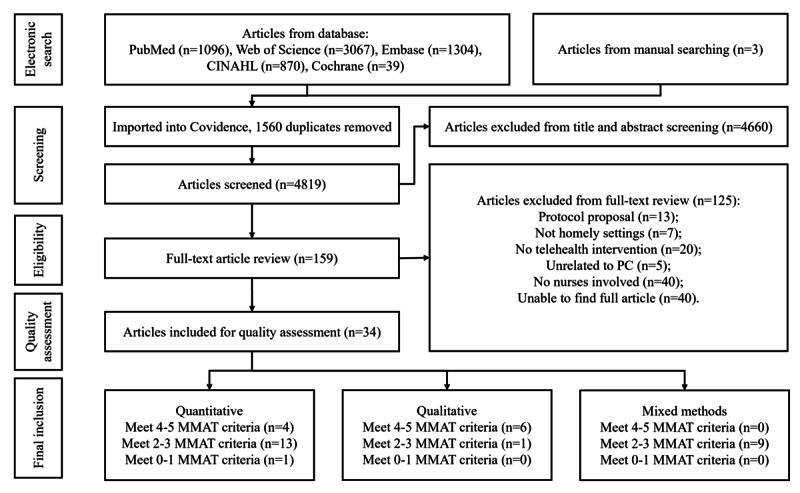
PRISMA (Preferred Reporting Items for Systematic Reviews and Meta-Analysis) flow diagram of systematic literature review. MMAT: Mixed Methods Appraisal Tool; PC: palliative care.

The electronic search was accomplished on May 14, 2024. We identified 4819 unique articles, and 1560 duplicates were removed on Covidence because they were repeated in different databases (Figures 1 and 2). We included a total of 34 articles, encompassing 29 unique primary research (Multimedia Appendix 3 [33-66]). The main items of the included studies are detailed in Table 2.

**Table 2:** Interested items of innovations of included studies.

Item of interest	Innovations (n=29)
Innovation deliverer	Nurses (n=8): A nurse (n=3) [43,44,56] Nursing teams (n=5) [46-48,55,63] Nurses and multiprofessional teams (n=18): Nurses and physicians (n=10): a nurse and a PC^a^ physician (n=3) [33,34,36,40,41,67]; a nurse or physician (n=2) [49,65]; PC or hospice nurses and physicians (n=4) [37,38,45,52,54,68]; PC nurses and specialist teams (n=1) [39] Nurses, physicians, and other health care providers (n=5): social workers [42,62,66], physical therapists [59,60,62], occupational therapists [62], and PC coordinators [51,62] Homecare nurses and a PC team (nurses and physicians) (n=4) [53,57,58,61]
Innovation recipient	Patients (n=13) [42,44,47-49,52,54-56,58,61,62,66,68] Family caregivers (n=2) [39,65] Patients and family caregivers (n=11) [33,34,36-38,40,41,43,45,46,51,53,59,60,63,67]
Innovation	Model of care: Telehealth only (n=21) [37-49,51,52,54-56,58-63,68] Initial in-person consultation and telehealth (n=3) [33,34,36,65,67] Simultaneous telehealth and in-person care (n=2) [53,66] Telehealth technologies: Telephone (n=11) [33,34,36,39,42,44,46,47,54,55,65-67] Phone- or web-based application (n=5) [37,38,45,48,52,56,68] Video consultation (n=7) [40,41,49,50,53,58-61] Phone call and video consultation (n=1) [63] Not explicitly stated (n=5) [43,51,57,62,64]
Setting and institution	Patients and their family caregivers receive services in home setting; nurses (and multiprofessional teams) are from various settings and institutions: Outpatient clinics (n=8) [33,34,36,39,42,44,46,49,56,67], such as palliative care clinics, specialist clinics, and community outreach clinics Hospice and palliative care organizations (n=7) [37,38,43,45,51,61,62,65], such as hospice agencies, palliative care service centers Specialist departments of hospitals and institutes (n=4) [54,55,59,60,66] Community organizations (n=3) [52,58,63,68] Homecare service organizations (n=1) [48] Emergency medical service organization (n=1) [47] Local health care system (n=2) [40,41,53] There are 3 included studies conducted in rural settings [33,34,53,58]
Outcome and measurement	Clinical outcomes: Patients: QOL^b^ (FACIT-Pal^c^ [33,56,67], KCCQ^d^ [42,56,67], FACT-G^e^ [42,47], CCQ^f^ [42], EORTC QLQ-C15-PAL^g^ [48], PROMIS-10^h^ [41]); symptom impact (QUAL-E^i^ [33], ESAS^j^ [41,47,49]); mood (CES-D^k^ [33], HADS^l^ [49,56,67], PHQ^m^ [42,44], GAD^n^ [42,44]); 1-year survival [33]; all-cause mortality [42]; global health [67]; pain [37,67]; loneliness (UCLA-3^o^ [47]); activation (PAM^p^) [48]; Palliative Care Outcomes Collaboration (PCOC^q^) [58] Family caregivers: QOL (CQOL-C^r^ [34], BCOS^s^ [36]); mood (CES-D [34], HADS [36]); caregiver burden (MBCB^t^ [34,36], ZBI^u^ [41]); caregiver preparedness (PCS^v^ [41], PROMIS-10 [41]); global health [36]; pain misconceptions [37] Nurses: telehealth readiness (TRAT-C^w^ [64]); innovative self-efficacy (ISES-C^x^ [64]) Medical resources use: hospital or intensive care unit days [33,67]; emergency department visits [33,52,56,67]; hospitalizations [67]; chemotherapy in last 14 days [33]; death location [33]; days at home in the last 6 months of life [41]; referrals to PC [44] Implementation outcomes: usability, feasibility, and acceptability of telehealth [38,46,52,55,56,58]; satisfaction of telehealth [48,55,58,66]; quality of conversation [38]; audiovisual quality [53]; completion of advance directives [38]; comfort level [45] Experiences and perspectives (interviews): Patients (n=9) [43,48,51,53,54,57,60,63,68] Family caregivers (n=9) [39,43,51,53,57,60,63,65,68] Nurses and other health care providers (n=10) [40,45,51,53,57,59,61,62,65,68]

^a^PC: palliative care.

^b^QOL: quality of life.

^c^FACIT-Pal: Functional Assessment of Chronic Illness Therapy Palliative Care.

^d^KCCQ: Kansas City Cardiomyopathy Questionnaire.

^e^FACT-G: Functional Assessment of Chronic Illness Therapy-General.

^f^CCQ: Clinical Chronic Obstructive Pulmonary Disease Questionnaire.

^g^EORTC QLQ-C15-PAL: European Organization for the Research and Treatment of Cancer Quality-of-Life Questionnaire Core 15-Palliative.

^h^PROMIS: Patient-Reported Outcomes Measurement Information System.

^i^QUAL-E: Quality of Life at End of Life.

^j^ESAS: Edmonton Symptom Assessment System.

^k^CES-D: Center for Epidemiologic Studies-Depression scale.

^l^HADS: Hospital Anxiety and Depression Scale.

^m^PHQ-8: Patient Health Questionnaire-8.

^n^GAD-7: Generalized Anxiety Disorder-7.

^o^UCLA-3: University of California, Los Angeles 3-item Loneliness Scale.

^p^PAM: Patient Activation Measure.

^q^PCOC: Palliative Care Outcomes Collaboration.

^r^CQOL-C: Caregiver Quality of Life Scale–Cancer.

^s^BCOS: Bakas Caregiver Outcomes Scale.

^t^MBCB: Montgomery-Borgatta Caregiver Burden scale.

^u^ZBI: Zarit Burden Interview.

^v^PCS: Preparedness for Caregiving Scale.

^w^TRAT-C: Telehealth Readiness Assessment Tools.

^x^ISES-C: Innovative Self-Efficacy Scale.

The included studies were conducted in the United States (n=11) [33-47,67,69], the Netherlands (n=3) [48-50], Canada (n=3) [51-53], Italy (n=2) [54,55], Iran (n=2) [56,57], Australia (n=1) [58], Denmark (n=1) [59,60], Switzerland (n=1) [61], Norway (n=1) [62], the United Kingdom (n=1) [63], China (n=1) [64], Lebanon (n=1) [65], and India (n=1) [66].

We included 6 qualitative studies—qualitative description (n=5) [43,51,57,62,65] and phenomenological methodology (n=1) [59,60]. A total of 17 qualitative studies were included—randomized clinical trials (n=8) [33,34,36-38,42,44,47,49,55,56,67], nonrandomized clinical trials (n=3) [39,54,55], cross-sectional studies (n=2) [50,64], and a prospective study (n=1) [66]. In addition, there were 9 studies with the design of mixed methods research—convergent parallel design (n=5) [45,53,58,61,63], explanatory sequential design (n=3) [40,41,48,52], and multiphases design (n=1) [46].

### Outcomes of Quality Assessment

In total, 10 articles met 4-5 MMAT criteria, 23 articles met 2-3 MMAT criteria, and 1 article met 0-1 MMAT criteria. A more detailed presentation of these studies’ quality assessment is in Multimedia Appendix 4 [33-66].

Most of the included qualitative studies applied qualitative description to explore the targeted question, rather than applying a specific methodology [43,51,62,65]. The most common comments were insufficient reports on interview guide design, such as question lists [62,65]. There was a study collecting data applying both individual interviews and focus groups, without reporting the integration of qualitative data [62].

The main quality comments of included randomized controlled trials (RCTs) focused on the random allocation method, blind methods, sample size, and intervention adherence. Some of the included RCTs did not explicitly report random allocation methods [41,44] and allocation concealment measures [37,47,56]. Some studies applied a nonblinded method [49] or a single-blinded method [33,37], risking performance bias, and detection bias. Outcome assessors were blinded in some RCTs, but participants and intervention providers were not [36,67]. Some RCTs did not describe blind methods [34,42,44], limiting research transparency. Some RCTs reported a reduced sample size [33], high attrition [49], and early termination [41], affecting the accuracy of the study and reliability. Some RCTs reported low adherence [37,67] or did not report complete adherence data [34,44,47]. The most common comments on non-RCTs were no adjustment for confounders in analysis and no adherence data [39,54,55]. As for the quantitative descriptive study, the sampling method was not described [38].

Most of the mixed methods research studies did not explicitly describe integration strategies and presented quantitative data and qualitative data separately [40,48,52,53,58,61,63]. There is also a study that did not address potential discrepancies between qualitative and quantitative findings [46].

### Roles of Nurses in Telehealth Home-Based Palliative Care

As the main delivers of telehealth home-based palliative care services delivery, nurses take various responsibilities, which are categorized by professional level and specialization level. According to the professional level, the nurses involved in these included studies were all registered nurses. On this basis, there were also advanced practice nurses (n=2) [33,34,43], nurse practitioner (n=3) [45,49,51], and clinical nurse specialist (n=1) [53]. Nurses could be categorized into 3 types based on specialization, that is, palliative care nurse (n=12) [39,43,46,49,51-54,58-62,66] or hospice nurse (n=2) [37,38,45], home care nurse (n=5) [40,41,48,53,61,66] or community nurse (n=2) [58,59], and specialist nurse (n=2) [55,62]. They undertook the responsibilities associated with the role of nurse coach (n=4) [33,34,36,44,46,67], nurse case manager (n=3) [40,41,54,55], and nurse coordinator (n=1) [62].

The contents of nurse-delivered, telehealth, home-based palliative care services involved assessment and screening (n=7) [33,34,36,39,44,46,51,53,67], palliative care consultation (n=13) [33,34,36,45,49,51,53,54,56,58-61,63,65,67], nursing coaching session (n=5) [33,34,36,42,44,46,67], regularly follow-up (n=7) [33,34,36-38,42,51,56,62,67], coordinating medical resources (n=12) [33,34,36,39,42,43,47,49,52,54,58,61,66,67], 24×7 services (n=2) [45,65], remote monitoring (n=4) [37,38,52,55,62], educational support (n=3) [39,47,48], technological support (n=5) [40,41,53,55,58,62], and home care (n=5) [40,41,48,53,61,66].

### Facilitators and Barriers to Implementing Nurse-Delivered Telehealth in Home-Based Palliative Care

**Figure 3:**
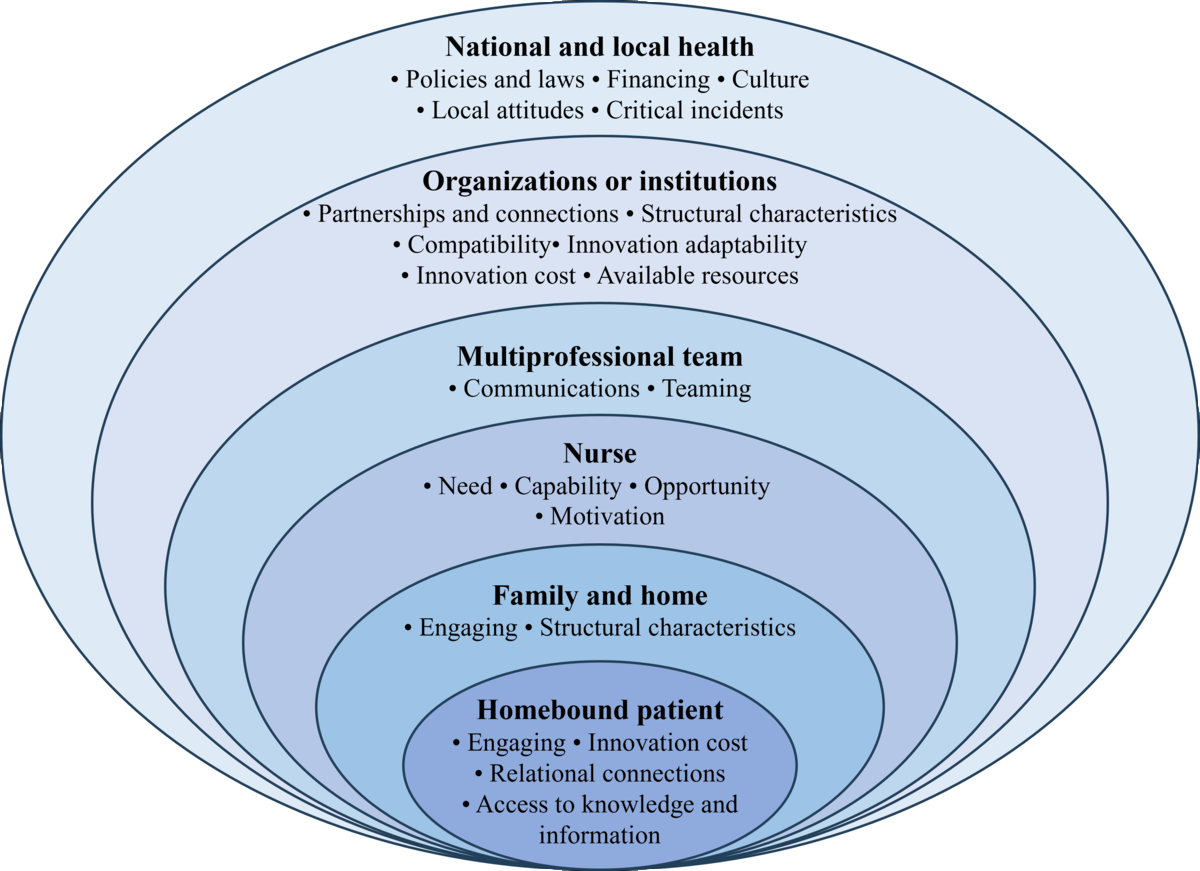
A multilevel framework of implementing nurse-delivered telehealth home-based palliative care.

Guided by CFIR 2.0, the qualitative data and the transformed quantitative data were integrated and synthesized to identify the facilitators and barriers in 6 implementation levels and 20 constructs for implementing nurse-delivered, telehealth, home-based palliative care, as detailed in Figure 3 and Table 3.

**Table 3:** Identified multilevel facilitators and barriers to implementation of telehealth home-based palliative care based on CFIR^a^ 2.0.

Construct of CFIR 2.0				Facilitators and barriers		Quotes
**National and local health level**						
	Policies and laws					
			Barrier: Lack of prescriptive authority for nurses		In our healthcare system (Iran), nurse prescribing is not legal…Therefore they cannot prescribe at home and manage symptoms of cancer patients [57].	
			Barrier: Lack of legal guarantee for home visits		Since the visit takes place at home in an informal place for caring, it can pose risks to the family and healthcare providers [57].	
	Financing		Barrier: Lack of reimbursement and payment mechanism		There is no health insurance for coverage of home healthcare services in our healthcare system (Iran) [57]. Ontario palliative fee-for-service billing codes provided significantly less compensation for virtual palliative care than in-person care in the home, thus incentivizing in-person visits [51].	
	Culture		Barrier: Culture building		In the public sight, there is no difference between care and treatment, and the hospital is the right place for both; a change in this mindset requires culture-building [57].	
	Local attitudes					
			Barrier: Ethical dilemmas in virtual care		There are things that hospice nurse could see on a one-on-one basis that they may not see on a screen [43].	
			Barrier: Insufficient awareness of the public		Public awareness about palliative care and home care is not enough [57].	
	Critical incidents		Facilitator: Impact of COVID-19 pandemic		They used AVA often during COVID to help keep families connected when visiting was prohibited [45].	
**Organizations and institutions level**						
	Partnerships and connections					
			Barrier: Lack of integration across telehealth systems and documentation systems		The most prominent challenge in implementing the RHC was the lack of integration across different healthcare systems and services in the documentation concerning the patient’s treatment and care [62].	
			Barrier: Poor transitional care from hospital to home		Poor transfer of care from the hospital to home care centers…Can be an obstacle to safe transfer from hospital to home [57].	
	**Structural characteristics**					
		Information technology infrastructure				
			Barrier: Limited reliable internet coverage		Some caregivers thought that rural network connectivity was unreliable which may represent a barrier to fully deploying telehealth in areas with less reliable network coverage [58].	
			Barrier: Technical problems		Due to the Low internet speed in Iran, we had an internet issue with most of the platforms, like kicking out of the room because of bad connection or not having a good and clear voice and video [56].	
		Work infrastructure				
			Facilitator: Technical maintenance personnel		The community nurses solved the technical problems together with the patient [59].	
			Barrier: Insufficient nurse staffing		Unfortunately, they (nurses) are not involved in the care at home and community in our society [57].	
	Compatibility		Facilitator: Compatibility with current workflow		The nurses use of technological devices like tablets is common in the community nurses’ daily clinical practice [59].	
	Innovation adaptability		Facilitator: Adapting to various settings		Thirty-one (77.5%) participants did not have difficulty adjusting to AVA in the patient’s home, and 18 (51.4%) did not have difficulty adjusting to AVA in extended care facilities [45].	
	Innovation cost		Facilitator: Cost avoidance to the health care system		Those clinicians agreed that with RELIEF, each patient was able to be managed in the home, and not only were emergency department visits prevented, but at least one admission to hospital was avoided in each case [52].	
	Available resources		Barrier: Lack of location to set up infrastructure		In my home care institutions, there would be no quiet computer workstations available [61].	
**Multiprofessional team level**						
	Communications		Facilitator: Communications between health care providers		The HCPs agreed that digital care conferences could increase understanding…and improve coordination and communication between professionals [61].	
	Teaming		Facilitator: Cooperations of nurses		The use of video consultations allowed the community nurses and the SPC team nurse to co-operate on an inter-professional level and supplement each other [59].	
**Nurse level**						
	Need					
			Facilitator: Self-worth recognition		I feel it has great value that I am sitting there [59].	
			Facilitator: Psychosocial support		Telehealth made me feel very supported during my home visits [58].	
			Facilitator: Professional development needs		I think I would’ve felt safer with courses or training before we jumped into it [RHC in palliative care] [62].	
	Capability					
			Facilitator: A higher level of telehealth readiness and innovation self-efficacy		The total score of TRAT-C was 65.31 ± 9.09 (range from 20 to 85), which indicated that Chinese palliative care specialist nurses had a moderate level of readiness to provide the telehealth services to patients [64].	
			Facilitator: Trained and qualified in palliative care		Most providers felt that support using telemedicine can only be provided safely by health providers who have extensive experience in palliative care [65].	
	Opportunity		Facilitator: Nurse leadership recognition in multiprofessional teams		The nurses preferred to have a nurse lead the conference (71.4%). The physicians also revealed a preference for a nurse leader (57.2%) [61].	
	Motivation					
			Facilitator: Work efficiency improvement		Using video visits increases my productivity (nurses: 3.0 [1.3]); using video visits makes it easier to do my job (nurses: 3.2 [1.3]) [40].	
			Barrier: Fear of death and anxiety		Most of the HCPs expressed their feelings of fear and insecurity regarding cancer and death. One informant believed that this was closely connected to the general perception of cancer as representing death, and to the hcps’ personal experiences and attitudes toward death [62].	
			Barrier: Additional scheduling burden		Physicians and nurses noted that video visits place an additional burden on nurses, who set up and facilitate video visits during their home visits. Scheduling is an ever-present challenge [40].	
			Barrier: Dissatisfied and concerned about exclusively virtual care		Providers described being uncomfortable and dissatisfied with providing exclusively virtual care to patients and their families since they were not able to conduct comprehensive patient assessments virtually and consequently felt they were not providing high-quality care [51].	
			Barrier: Limited ability to form connections with patients		Virtual palliative care limited their ability to form personal connections with patients and their caregivers [51].	
**Family and home level**						
	**Engaging**					
		Innovation deliverers	Facilitator: Reliable family caregivers		It is always helpful to have a caregiver at home who is reliable and communicates well with the medical team [65].	
		Innovation recipients	Facilitator: Equitable involvement of family		The relatives could join the video consultations on equal terms with the patients and their involvement was initiated through the App development [59].	
	**Structural characteristics**					
		Physical infrastructure	Barrier: None available device		Six patients were excluded due to a lack of electronic skills, and no tablets were available in time [59].	
**Homebound patient level**						
	**Engaging**					
		Innovation recipients				
			Facilitator: Technical assistance		It’s okay if we have technical assistance [43].	
			Facilitator: Existential value of telehealth services		The service reduced their fear of abandonment, sense of isolation, and uncertainty and could have improved the patient’s sense of control [55].	
			Barrier: Lack of telehealth competency		Six patients were excluded due to a lack of electronic skills, and no tablets were available in time [59]. Patients and caregivers who reported never using computers had the lowest CAS scores [38].	
			Barrier: High age and heavy symptom burden		Some participants found the use of the technology during the telehealth visit to provide a disjointed experience and felt their age decreased their comfort of using telehealth [43]. I don’t think the tablet will help me later on as I’m getting worse. At that time, it will be the community nurses who take over because I will have more physical needs. Some things cannot be taken care of on a tablet [60].	
			Barrier: Privacy and information safety concerns		I think in some ways the separation, there was an element of control because what we wanted was privacy, so bringing somebody else [in] then would be an invasion of your privacy [63].	
	Innovation cost					
			Facilitator: Time and cost savings		Many participants found the telehealth visit to be more efficient and convenient for them personally, requiring less energy spent getting dressed, traveling to the provider's office, and waiting for the appointment [43].	
			Barrier: Long timing in telehealth visits		The longer you look at a computer screen or a phone screen, you get tired [43].	
	Relational connections		Facilitator: Trust-based nurse-patient relationship		When adequate trust is established with a care provider, struggles with technology are more tolerable [43]. They (the patients) pay attention and I feel that we build a trustworthy relationship [59]. Over time, patients and carers got to know the nurses and developed rapport and trust [63].	
	Access to knowledge and information		Facilitator: Patient education		30 (70.0%) participants suggested patient education (videos, instruction sheets) would be helpful [45].	

^a^CFIR: Consolidated Framework for Implementation Research.

#### National and Local Health Systems: Disparities Between High-Resource and Low-Resource Regions

Globally, critical incidents significantly impact the development of telehealth. For instance, the isolation policies during the COVID-19 pandemic greatly facilitated the development of home-based palliative care delivered via telehealth [50,51,54,57,64]. Overall, the implementation of nurse-delivered, telehealth, home-based palliative care is profoundly influenced by the disparities in national and local health systems between high-income and middle- to low-income settings. For example, in high-income countries and regions such as the United States and Canada, laws grant prescription authority to nurse practitioners, enabling nurses to participate in patient medication management. In contrast, in countries and regions with middle to low levels of medical resources, laws concerning nurse home visit safety are yet to be fully established, which hinders the progress of telehealth home-based palliative care [57,61]. Financing is also an important factor affecting the development of telehealth home-based palliative care. The lack of inclusion of homecare services in the health insurance system [57] or the fact that telehealth home-based palliative care services are not as well supported as in-person care services [51] are barriers. Furthermore, due to ethical dilemmas in telehealth and insufficient awareness of the public, local negative attitudes also present barriers [43,57,70].

#### Organizational and Institutional Systems: Inadequate Integrative Telehealth Service Delivery

At the organization and institution level, the most significant challenges are the partnerships and connections between different organizations. Barriers include inadequate integrative telehealth system and documentation system [62] and poor transitional care from hospital to home [57]. Of the structural characteristics, information technology infrastructure (such as unreliable internet coverage [58] and technical problems [56]) and work infrastructure (such as insufficient nurse staffing) are the barriers to implementing telehealth home-based palliative care. However, community nurses working with patients to solve technical problems alleviated some of these difficulties [59]. The lack of a location to set up telehealth infrastructure also hinders nurses from providing home-based palliative care services via telehealth technologies [61]. The compatibility with current workflow [59], adaptability to various settings [45], and cost avoidance to the health care system [52] are the facilitators to implementing nurse-delivered, telehealth, home-based palliative care services.

#### Multiprofessional Team and Health Care Providers: Effective Coordination and Teaming

At the level of a multiprofessional team, communications between health care providers [61] and the cooperation of nurses [59] are the most important factors in implementing nurse-delivered, telehealth, home-based palliative care. In the studies included in this review, home-based palliative care services involve palliative care teams from various medical institutions related to referrals, nursing teams that provide phone call services, and teams that offer homecare services. Telehealth such as videoconferences could increase understanding and cooperation between multiprofessional physicians and nurses. Effective communication ensures seamless coordination and continuity of care, enabling all parties involved to understand the patient’s needs and preferences, share medical information accurately, and adjust care plans as necessary. This collaboration is essential for providing comprehensive support that addresses both the physical and psychosocial needs of patients and their families within the home setting [59,61].

#### Nurses: Positive Professional Identity and Continuous Professional Development

Nurses have a need for self-worth recognition [59], psychosocial support [58], and professional development [62]. A good telehealth home-based palliative care system can meet the needs of nurses. Nurses with higher levels of telehealth readiness, innovation self-efficacy, and training in palliative care have the capability to deliver telehealth home-based palliative care [64,65]. Telehealth home-based palliative care also provides nurses with opportunities to develop nurse leadership in multiprofessional teams [61]. On the construct of motivation, telehealth technologies improve the work efficiency of nurses, which could be the facilitator to implement home-based palliative care [40]. However, due to the fear of death and anxiety [62], additional scheduling burden [40], dissatisfaction with exclusively virtual care [51], and concerns about the limited ability to form connections with patients [51], nurses reduce their willingness to deliver telehealth home-based palliative care.

#### Family and Home: Family Caregivers of Both Deliverers and Recipients

Family caregivers play an integral role in supporting both the delivery and receipt of telehealth home-based palliative care services. Reliable family caregivers contribute positively by assisting patients in managing their care and navigating the technology required for remote consultations [65]. Equal involvement in videoconferencing also facilitates family engagement [59]. However, the lack of physical infrastructure in home settings limits the engagement of family caregivers and patients [59]. This level underscores the necessity of considering the socioeconomic context of family units when planning and delivering telehealth home-based palliative care services.

#### Homebound Patient: Conflicted Patients and Personalized Patient Needs

At the level of the patient who is homebound, privacy concerns [63], lack of telehealth competency [38,59], advanced age [43], and heavy symptom burden [60] are the main barriers to reducing the patients’ engagement. Technical assistance [43] and the existential value of telehealth [55] play a role in promoting the engagement of patients. Telehealth home-based palliative care services could save some patients’ time and cost to travel to the hospital [43]. However, long timing in telehealth visits makes some patients uncomfortable [43]. Patients and nurses could build trust-based relationships via telehealth technologies [59,63]. The relational connection between patients and nurses, and patient education enhance the acceptability of care [45].

## Discussion

### Principal Findings

By synthesizing quantitative and qualitative data, this integrative systematic review identified the roles of nurses in telehealth home-based palliative care and synthesized the multilevel factors to implement nurse-delivered, telehealth, home-based palliative care. Building on these findings, we have conducted a comprehensive analysis to formulate evidence-based recommendations for future implementation.

With the development of telehealth technologies, the places of palliative care services have extended to the home setting, and the roles of nurses have consequently changed [71]. This presents new challenges for future nursing competencies and practice. Due to the shortage of specialized palliative care staffing in home-based palliative care, we find that nurses’ responsibilities partially overlap with those of physicians, medical social workers, and technical personnel. Research suggests that ambiguous professional boundaries may increase the complexity of nursing practice and create role ambiguity, which may influence nurses’ own professional identity [72]. However, with telehealth-enabled empowerment, nurses could actively rebuild a stronger professional identity through fluid role boundaries [73] and facilitate communications and cooperation with multiprofessional teams [59,61]. With a firm professional identity, the nurses still can develop new roles even if the existing role collapses [74]. Similarly, a systematic review and meta-analysis found that developing the intrinsic motivation of nurses such as professional identity development could improve nurses’ job satisfaction effectively, which could reduce turnover of nurses [75].

The formation of nurses’ professional identity is a continuous process that occurs throughout nurses’ careers from nursing students’ education to continuing education [74]. It is suggested that palliative care training should be integrated into nurse education, including nursing students, primary care nurses, and specialist palliative care nurses [76]. A survey has shown that nursing students’ professional identity is related to death anxiety, and palliative care education could help them relieve death fear and develop a higher professional identity [77]. It is consistent with our findings that after receiving professional palliative care training, improvements in the capability of empathy, communication skills, and self-care techniques could help nurses reflect on and negotiate conflicts within their roles [62]. In addition to palliative care competence, we find that telehealth competence is also important for nurses to deliver telehealth home-based palliative care. One of the included studies indicated that the degree of innovation in self-efficacy of palliative care nurses significantly affects their telehealth readiness, and nurses with higher levels of innovation self-efficacy and telehealth readiness have a stronger willingness and motivation to apply telehealth technologies in home-based palliative care [64]. The telehealth competence of nurses also includes the ability to evaluate patients’ needs and willingness to use telehealth services, use telehealth devices and applications, solve technical problems, interact with patients, and improve their competence constantly [71]. A comprehensive telehealth training system should be established for palliative care specialist nurses [64,78].

Although nurses make up the largest group of health care providers in many countries and regions [79], there is a point of review that the nursing discipline has insufficiently communicated nurses as influential leaders [80]. We find that palliative care nurses are recognized and given the chance to lead the delivery of telehealth home-based palliative care in both nursing teams and multiprofessional teams [81]. The professional identity of palliative care nurses is further established in interpersonal communication and teaming among professional health care providers [74,81]. We find that nurses are deeply involved in all aspects of telehealth home-based palliative care implementation. In some countries and regions with low resources, nurses could advocate for nurses’ prescriptive authority at the policy and legal level, to improve the accessibility of medication management for patients in telehealth home-based palliative care [18]. With the transformative development of telehealth technologies, nurses could create nurse-led implementation solutions and influence actions via telehealth in multilevel settings, including nursing practice, academic research, clinical management, policy promotion, and public health in home-based palliative care [80,82]. Furthermore, nurses could lead the development of nursing education, standard of care, clinical guidance, implementation manual, quality control, and evaluation systems for the application of telehealth technologies in home-based palliative care services to improve patient safety [19,83]. As telehealth delivers, nurses also provide valuable contributions to the interdisciplinary cooperation of nursing and telehealth [84].

The core of palliative care is consistent with the orientation of the nurse-led model of care, which is to deliver patient-centered holistic care [19]. The professional identity of nurses is also established in the trust-based nurse-patient relationship [74]. Telehealth technologies transform the nurse-patient interaction [71]. Some nurses are concerned that virtual palliative care limits their ability to form personal connections with patients and their caregivers, due to lack of physical contact and incomplete assessment [51]. It also reflects an ethical criticism that telehealth home-based palliative care lacks humanity [70]. However, our review finds that from the perspectives of patients, telehealth seems to have the potential to promote the building of trust-based nurse-patient relationships [59,63]. First, nurse-delivered care could relieve patients’ symptom burden and improve their quality of life, indicating that the professional competence of nurses in palliative care could increase patient trust [15]. Second, patients who are homebound could seek timely assistance from nurses through telehealth technologies when they encounter acute symptoms, which relieves patients’ existential anxiety, thus promoting their trust in nurses [55]. Third, with the emotional and technical support of nurses, patients are empowered with self-efficacy in telehealth [43]. Furthermore, nurses also empower the patients to engage in self-care and enable their family caregivers to be equally involved in home care through health education [18]. In conclusion, with the transformation of the nurse-patient relationship, nurses need competence in interacting with patients by using telehealth technologies [71].

This study explores facilitators and barriers influencing nurse-delivered, telehealth, home-based palliative care services from an implementation science framework, without evaluating intervention effectiveness. The included studies used multidimensional outcome measures, encompassing clinical outcomes, health care resource utilization, implementation outcomes, and experiences (patients, caregivers, nurses, and other stakeholders). Among these, quality of life and mood emerged as the most frequently assessed outcomes for patients and family caregivers. Future studies could employ systematic review and meta-analysis to examine the effectiveness of innovations.

### Limitations

Heterogeneity in outcome measures limiting comparability. Furthermore, the lack of racial and ethnic diversity limited the generalizability of the study. There are also potential biases from including only English studies, which might affect the comprehensiveness.

### Conclusions

This integrative systematic review synthesizes evidence on nurses’ evolving roles in telehealth home-based palliative care and identifies multilevel facilitators and barriers to nurse-delivered, home-based palliative care implementation. With the empowerment of telehealth technologies, nurses could establish a stronger professional identity and develop leadership in home-based palliative care. Systematic palliative care and telehealth education and training are critical across the nurses’ professional development, which facilitates to build nurses’ competence in home-based palliative care and develop trust-based nurse-patient relationship. Nurses are supposed to leverage influence to promote nursing practice, clinical management, and policy support in the implementation of telehealth home-based palliative care.

## Appendix

Multimedia Appendix 1 PRISMA (Preferred Reporting Items for Systematic Reviews and Meta-Analyses) 2020 checklist of this integrative systematic review.

Multimedia Appendix 2 The search strategy of PubMed, Web of Science, and Embase.

Multimedia Appendix 3 Characteristics of the included studies based on TIDieR (Template for Intervention Description and Replication) checklist.

Multimedia Appendix 4 Assessment of methodological quality of the included studies using Mixed Methods Appraisal Tool (version 2018).

## Abbreviations

CFIR: Consolidated Framework for Implementation Research
JBI: Joanna Briggs Institute
MMAT: Mixed Methods Appraisal Tool
PRISMA: Preferred Reporting Items for Systematic Reviews and Meta-Analysis
RCT: randomized controlled trial
SPIDER: Sample, Phenomenon of Interest, Design, Evaluation, and Research type
TIDieR: Template for Intervention Description and Replication
