# Supplementary material for: Nurse-Delivered Telehealth in Home-Based Palliative Care: Integrative Systematic Review
Source: J Med Internet Res. 2025 May 5;27:e73024. doi: 10.2196/73024 (PMC12089877; doi:10.2196/73024)
Supplement: Multimedia Appendix 2 [file jmir_v27i1e73024_app2.docx]

**PubMed, 1096**

1. (telemedicine[MeSH Terms]) OR (telemedicine[Text Word]) (52,835)
2. tele medicine[Text Word] OR telehealth[Text Word] OR tele health[Text Word] OR telecare[Text Word] OR tele care[Text Word] OR telecommunicat*[Text Word] OR tele communicat*[Text Word] OR teleconferenc*[Text Word] OR tele conferenc*[Text Word] OR teleconsultat*[Text Word] OR tele consultat*[Text Word] OR telenurs*[Text Word] OR tele nurs*[Text Word] OR telemonitor*[Text Word] OR tele monitor*[Text Word] OR teletherap*[Text Word] OR tele therap*[Text Word] OR telerehab*[Text Word] OR tele rehab*[Text Word] (40,956)
3. "ehealth"[Text Word] OR "e-health"[Text Word] OR "e-health"[Text Word] (12,468)
4. "mhealth"[Text Word] OR "m-health"[Text Word] OR "m-health"[Text Word] (11,471)
5. "remote health"[Text Word] OR "remote care"[Text Word] OR "remote medicine"[Text Word] OR "remote nurs*"[Text Word] OR "remote communicat*"[Text Word] OR "remote therap*"[Text Word] OR "remote consult*"[Text Word] OR "remote conference*"[Text Word] (8,859)
6. "video conferenc*"[Text Word] OR "videoconferenc*"[Text Word] (7,067)
7. "digital health"[Text Word] OR "digital care"[Text Word] (10,120)
8. #1 OR #2 OR #3 OR #4 OR #5 OR #6 OR #7 (106,302)
9. "palliative care"[MeSH Terms] OR "palliative care"[Text Word] (81,985)
10. "palliative medicine"[MeSH Terms] OR "palliative medicine"[Text Word] (3,049)
11. "hospice and palliative care nursing"[MeSH Terms] OR "hospice and palliative care nursing"[Text Word] (2,638)
12. "terminal care" [MeSH Terms] OR "terminal Care" [Text Word] (59,316)
13. "hospice care" [MeSH Terms] OR "hospice care" [Text Word] (10,736)
14. "advance care planning" [MeSH Terms] OR "advance care planning" [Text Word] (13,489)
15. "advance directives" [MeSH Terms] OR "advance directives" [Text Word] (9,254)
16. "end of life care"[Text Word] OR "eol care"[Text Word] (15,665)
17. "pain manage*"[Text Word] OR "symptom manage*"[Text Word] (76,458)
18. "mental health"[Text Word] OR "mental disorders"[Text Word] OR "mental stress"[Text Word] OR "psychological health"[Text Word] OR "psychological stress"[Text Word] (475,278)
19. "bereavement support"[Text Word] OR "social support"[Text Word] OR "spiritual support"[Text Word] (112,370)
20. #9 OR #10 OR #11 OR #12 OR #13 OR #14 OR #15 OR #16 OR #17 OR #18 OR #19 (765,297)
21. "nurse"[MeSH Terms] OR "nurs*"[Text Word] (833,096)
22. #8 AND #20 AND #21 (1096)

**Web of Science Core Collection, 3067**

1. TS=(telemedicine) (28,596)
2. TS=(telehealth) (14,651)
3. TS=(telecare) (1,278)
4. AB=(tele medicine) (361)
5. AB=(tele health) (1,210)
6. AB=(tele care) (1,375)
7. AB=(telecommunicat*) OR AB=(tele communicat*) (23,042)
8. AB=(teleconferenc*) OR AB=(tele conferenc*) (1,597)
9. AB=(teleconsultat*) OR AB=(tele consultat*) (1,760)
10. AB=(telenurs*) OR AB=(tele nurs*) (384)
11. AB=(telemonitor*) OR AB=(tele monitor*) (2,301)
12. AB=(teletherap*) OR AB=(tele therap*) (1,001)
13. AB=(telerehab*) OR AB=(tele rehab*) (1,558)
14. AB=(ehealth) OR AB=(e-health) OR AB=(e health) (151,618)
15. AB=(emedicine) OR AB=(e-medicine) OR AB=(e medicine) (34,907)
16. AB=(mhealth) OR AB=(m-health) OR AB=(m health) (86,223)
17. AB=(remote health) OR AB=(remote care) OR AB=(remote medicine) OR AB=(remote nurs*) OR AB=(remote communicat*) OR AB=(remote therap*) OR AB=(remote consult*) OR AB=(remote conference*) (38,813)
18. AB=(videoconferenc*) OR AB=(video conferenc*) (5,914)
19. AB=(digital health) OR AB=(digital care) OR AB=(digital medicine) (29,844)
20. OR/1~19 (374,346)
21. TS=(palliative care) (51,240)
22. TS=(palliative medicine) (8,082)
23. TS=(hospice and palliative care nursing) (1,274)
24. TS=(terminal care) (9,846)
25. TS=(hospice care) (11,602)
26. TS=(advance care planning) (5,887)
27. TS=(advance directives) (4,665)
28. AB=(end-of-life care) OR AB=(eol care) (14,585)
29. AB=(pain manage*) OR AB=(symptom manage*) (104,483)
30. AB=(mental health) OR AB=(mental disorders) OR AB=(mental stress) OR AB=(psychological health) OR AB=(psychological stress) (253,654)
31. AB=(bereavement support) OR AB=(social support) OR AB=( spiritual support) (101,564)
32. OR/21~31 (495,620)
33. TS=(nurs*) (325,672)
34. #20 AND #32 AND #33 (3,067)

**Embase, 1304**

1. 'telehealth'/exp (94,316)
2. 'telecommunication'/exp (125,429)
3. telemedicine:ab,ti OR 'tele medicine':ab,ti OR telehealth:ab,ti OR 'tele health':ab,ti OR telecare:ab,ti OR 'tele care':ab,ti OR telecommunicat*:ab,ti OR 'tele communicat*':ab,ti OR teleconferenc*:ab,ti OR 'tele conferenc*':ab,ti OR teleconsultat*:ab,ti OR 'tele consultat*':ab,ti OR telenurs*:ab,ti OR 'tele nurs*':ab,ti OR telemonitor*:ab,ti OR 'tele monitor*':ab,ti OR teletherap*:ab,ti OR 'tele therap*':ab,ti OR telerehab*:ab,ti OR 'tele rehab*':ab,ti (58,381)
4. ehealth:ab,ti OR 'e health':ab,ti OR emedicine:ab,ti OR 'm medicine':ab,ti OR 'e medicine':ab,ti OR mhealth:ab,ti OR 'm health':ab,ti (16,556)
5. 'remote health':ab,ti OR 'remote care':ab,ti OR 'remote medicine':ab,ti OR 'remote nurs*':ab,ti OR 'remote communicat*':ab,ti OR 'remote therap*':ab,ti OR 'remote consult*':ab,ti OR 'remote conference*':ab,ti (3,558)
6. 'video conferenc*':ab,ti OR videoconferenc*:ab,ti (8,147)
7. 'digital health':ab,ti OR 'digital care':ab,ti (7,440)
8. OR/1~7 (153,323)
9. 'palliative care'/exp (149,961)
10. 'palliative nursing'/exp (1,766)
11. 'terminal care'/exp (89,820)
12. 'living will'/exp (10,302)
13. 'advance care planning'/exp (7,140)
14. 'end-of-life care':ab,ti OR 'eol care':ab,ti (21,082)
15. 'pain manage*':ab,ti OR 'symptom manage*':ab,ti (61,462)
16. 'mental health':ab,ti OR 'mental disorders':ab,ti OR 'mental stress':ab,ti OR 'psychological health':ab,ti OR 'psychological stress':ab,ti (355,379)
17. 'bereavement support':ab,ti OR 'social support':ab,ti OR 'spirit* support':ab,ti (69,201)
18. OR/9~17 (684,332)
19. 'nurse'/exp (228,425)
20. nurs*:ab,ti (654,537)
21. OR/19~20 (708,133)
22. 8 AND 18 AND 21 (1304)
