# Supplementary material for: Nurse-Delivered Telehealth in Home-Based Palliative Care: Integrative Systematic Review
Source: J Med Internet Res. 2025 May 5;27:e73024. doi: 10.2196/73024 (PMC12089877; doi:10.2196/73024)
Supplement: Multimedia Appendix 3 [file jmir_v27i1e73024_app3.docx]

| **Brief name and country** | **Author and publication year** | **Why (aim)** | **Study design and methodology** | **How (telehealth)** | **Who delivered** | **To whom** | **Where** | **When and how** | **How well (outcomes and measurements)** | **Main findings** |
| --- | --- | --- | --- | --- | --- | --- | --- | --- | --- | --- |
| ENABLE III, USA | Bakitas et al. 2015 [1] and Dionne-Odom et al. 2015 [2] | To investigate the effect of early versus delayed PC. | quantitative study: a fast-track randomized clinical trial | in-person consultation and telephonic coaching | an APN as nurse coach and a PC clinician | 207 patients with advanced cancer and 122 family caregivers | Patients’ homes, a National Cancer Institute cancer center, a Veterans Affairs Medical Center, and 2 community outreach clinics in a rural setting | an in-person PC consultation, structured PC telehealth nurse coaching sessions | Patient: QOL (FACIT-Pal), symptom impact (QUAL-E), mood (CES-D), 1-year survival; family caregivers: QOL (CQOL-C), depression (CES-D), burden (MBCB); medical resources use: hospital/intensive care unit days, emergency room visits, chemotherapy in last 14 days, and death location | Early-entry participants’ survival 1-year after enrollment was improved compared with those who began 3 months later. Early-group CGs had lower depression scores at 3 months and lower depression and stress burden in the terminal decline analysis. |
| ENABLE CHF-PC, USA | Bakitas et al. 2020 [3] and Dionne-Odom et al. 2020 [4] | To determine the effect of an early PC telehealth intervention over 16 weeks of patients. | quantitative study: a randomized clinical trial | in-person consultation and telephonic coaching | a nurse coach and a PC clinician | 415 patients and 158 caregivers | Patients’ home and outpatient heart failure clinics | an in-person PC consultation, structured PC telehealth nurse coaching sessions | Patient: QOL (KCCQ, FACIT-Pal-14), global health, mood (HADS), pain; family caregiver: QOL (BCOS), mood (HADS), caregiver burden (MBCB), global health; medical resource use: hospital days and emergency department visits | The ENABLE CHF-PC treatment group showed significant improvements in heart health and QOL after 16 weeks compared to the control group. |
| PAINRelieveIt® and PAINReportIt®, USA | Wilkie et al. 2020 [5] and Schoppee et al. 2020 [6] | To compare effects of usual hospice care and PAINRelieveIt® on pain outcomes in patients and their lay caregivers, and examine computer use acceptability scores of end-stage cancer patients in hospice and their caregivers. | QUAN: a stepped-wedge randomized controlled trial and a survey | Application | hospice nurses and physician | 234 patients and 231 lay caregivers | Patients’ homes and not-for-profit hospice agency | daily follow-up for 7 days | Patient: pain (analgesic adherence, worst pain intensity, satisfaction), pain misconceptions; caregiver: pain misconceptions; implementation outcomes: use acceptability of PAINRelieveIt® | Experimental group had fewer misconceptions about pain (mean difference 0.38). Computer Acceptability scores were significantly associated with age and with prior computer use for both patients and caregivers. |
| ADAPT, USA | Bekelman et al. 2024 [7] | To determine the effect of a nurse and social worker palliative telecare team on QOL in outpatients compared with usual care. | QUAN: a randomized clinical trial | Telephone | nurses and social workers, and specialists | 306 outpatients (154 to the ADAPT intervention and 152 to usual care) | Patients’ homes and 2 Veterans Administration health care systems, and community-based outpatient clinics | 6 nursing phone calls | Patient: QOL (FACT-G), disease-specific health status (KCCQ, CCQ), depression (PHQ-8), anxiety (GAD-7), all-cause mortality; medical resource use: hospitalizations | After 6 months, the intervention group showed a 6-point increase in FACT-G score, compared to 1.4 points in the control group, a significant difference of 4.6 points. The intervention also significantly improved COPD and HF health status, as well as reduced depression and anxiety. |
| the Netherlands | Hoek et al. 2017 [8] | To determine the effect of weekly teleconsultations from a SPCT team improved symptom burden compared to “care as usual” | QUAN: a randomized clinical trial | video conference | the SPCT (a nurse, NP or physician) | 74 patients with advanced cancer | outpatient clinics of a tertiary university hospital and regional home care organizations | weekly teleconcultations | Patient: symptom burden (ESAS), mood (HADS) | Weekly teleconsultations added to usual PC for home-dwelling advanced cancer patients led to worse symptom scores. |
| Goc, USA | Doorenbos et al. 2016 [9] | To determine the effects of GoC intervention compared to usual care. | QUAN: a randomized clinical trial | telephone | a nurse coach | 80 HF patients | a HF outpatient clinic in an academic medical center | a telephone-based pre-visit coaching, a GoC discussion at the next upcoming HF clinic visit | Patient: depression (PHQ-9), anxiety (GAD-7); medical resource use: number of referrals to PC; implementation outcomes: number of GoC conversations, quality of communication, completion of advance directives | The GoC intervention resulted in more GoC conversations and higher quality communication between HF patients. |
| Iran | Mirshahi et al. 2024 [10] | To determine the feasibility and acceptability of the intervention. | QUAN: a pilot randomized controlled trial | WhatsApp, social platform | a nurse | 50 HF patients | a heart failure clinic | 6 weekly educational webinars and concurrent WhatsApp® group activities, with 6 weeks of follow‑up. | Patient: QOL (PKCCQ, FACIT-Pal-14), mood (HADS); medical resource use: emergency department visits; implementation outcome: feasibility and acceptability | This intervention is feasible and acceptable for Iranian heart failure patients with NYHA Class II/ III HF. |
| EMPallA, USA | Schmucker et al. 2021 [11] | To compare the effectiveness of specialty outpatient versus telephonic palliative care of older adults. | QUAN: a randomized clinical trial | telephonic case management | nurse-led team | 500 patients with advanced illness | 18 emergency department sites | NA | Patient: QOL (FACT-G), symptom burden (ESAS-r), loneliness (UCLA-3) | Our program demonstrated high rates of engagement, ACP, and hospice enrollment. |
| Telesupport, Italy | Vitacca et al. 2019 [12] | To test the feasibility of, and patient satisfaction with, an advanced care plan for severe COPD patients. | QUAN: a single-arm feasibility study | telephone | specializes nurse tutors as case manager | 10 COPD patients | Respiratory Rehabilitation Unit | an in-person PC consultation, monthly telephone monitoring for 6 months | Implementation outcomes: feasibility and satisfaction | The palliative talk was feasible and anxiety low during the talk. All patients expressed a high level of satisfaction of the service. |
| FamilyStrong, USA | Dionne-Odom et al. 2021 [13] | To examine first-year experiences of a nurse-led telehealth support service (FamilyStrong). | QUAN: a nRCT | telephone | PC nurses and neuro-oncology team | 53 family caregivers | oncology clini | NA | the first-year experiences of a nurse-led telehealth support service (FamilyStrong) | The most common problems caregivers wanted assistance with included: managing their relative’s health condition and symptoms (51%), coordinating care/services (21%), and planning for the future/advance care planning (17%). |
| Italy | Valenti et al. 2022 [14] | To present experience of a nursing telephone consultation service. | QUAN: a nRCT | telephone | PC nurses as case manager and physicians | 171 patients | a cancer institute | NA | experience of a nursing telephone consultation service | The majority (80.8%) were from patients followed at the outpatient clinic and the most common requests were for pain management (38.4%) and for updates on the clinical situation (23.8%). Other frequent requests were for medication management (18.9%) and scheduling (18.3%). |
| India | Balasubramanian et al. 2021 [15] | To find out the level of satisfaction of patients receiving e-palliative homecare (e-PHC). | QUAN: a prospective study | homecare and telephone consultation | e-PHC team (a PC nurse, a nursing assistant, a social worker and physicians in hospital) and homecare nurses | 120 patients | a tertiary Cancer Center | NA | Patient satisfaction (the validated e-Palliative Patient Satisfaction Questionnaire –Malayalam [PSQM]) | The mean score of response for General satisfaction, Technical quality, Communication, Financial aspect, Time spent with doctor and Accessibility and convenience were observed as 4.52, 3.92, 4.48, 4.55, 4.52 and 4.49 respectively. The overall satisfaction was found to be 4.39. |
| the Netherlands | Evering et al. 2022 [16] | To explore the intention to use video communication by HCPs in interprofessional terminal care and predictors herein. | quantitative study: a cross-sectional study | NA | NA | NA | Setting: Online survey  Sample: 90 first‑line HCPs involved in terminal care (at home, in hospices and/ or nursing homes) | NA | experience with video communication and constructs of intention to use | HCPs intend to use video communication in terminal care. Outcome expectancy and social influence are key predictors. |
| China | Guo et al. 2023 [17] | To examine TH readiness and its related factors among Chinese PC specialist nurses. | quantitative study: a cross-sectional study | NA | NA | NA | Setting: Chinese Nursing Association (CNA) Palliative Care Nurse Specialist Training Base;  Sample: 409 Chinese PC specialist nurses | NA | telehealth readiness (Telehealth Readiness Assessment Tool [TRAT]) and innovative self-efficacy (Innovative Self-Efficacy Scale [ISES]) | Telehealth readiness in nursing is influenced by experience with platforms, willingness to provide services, and innovative self-efficacy. |
| DSP, Lebanon | Salem et al. 2020 [18] | To assesse provider and caregiver perceptions of the safety and efficacy of the Distance Support Program (DSP). | QUAL: qualitative description | in-person encounter and 24/7 phone call services | a nurse or physician | 31 family caregivers | Balsam, the Lebanese Center for Palliative Care | one face-to-face encounter, 24/7 phone call services | provider and caregiver perceptions of the safety and efficacy of the Distance Support Program (DSP) | Caregivers valued DSP's phone support, though preferring home visits. Health providers found DSP efficient, safe with experienced staff, reliable access, and prior assessment. Clear phone communication with patients and caregivers was emphasized. |
| Denmark | Funderskov et al. 2019 (1) [19] and Funderskov et al. 2019 (2) [20] | To explore the advantages and disadvantages of using video consultations. | QUAL: phenomenological methodology | video consultation | a SPC team (physicians, nurses, and physiotherapists) and 5 community nurses | 11 patients and 3 relatives | Department of Oncology, Odense University Hospital | weekly video consultations | the advantages and disadvantages of using video consultations; the use of video consultations | Video consultations in specialized palliative home care are feasible. |
| Canada | Vincent et al. 2022 [21] | To explore the experiences and perceptions of community PC providers, patients and caregivers of HBPC during the COVID-19 pandemic. | QUAL: qualitative description | virtual encounter | 2 PC NPs, 13 PC physicians, and 3 PC coordinators | 5 patients and 14 caregivers | the Regional Palliative Care Consultation Team, the Orleans Palliative Care Team in Ottawa, the Temmy Latner Centre for Palliative Care | NA | the experiences and perceptions of community PC providers, patients and caregivers | Participants preferred in-person PC but saw virtual care as a useful supplement. |
| Norway | Oelschlägel et al. 2021 [22] | To explore municipal health-care professionals’ experiences associated with implementing a technological solution named “remote home care” in palliative home care for patients with cancer. | QUAL: qualitative description | welfare technology | the RHC service team (specialized nurses as cancer care coordinator, nurses, social worker, physical therapist, and occupational therapists) | patients with cancer | a RHC service center | NA | municipal health-care professionals’ experiences | Three themes were identified: 1) shifting from objective measures to assessing priorities for patients, 2) lack of experience and personal distress regarding cancer inhibits professional care, and 3) prominent organizational challenges undermine the premise of remote home care. |
| CPATH, USA | Bethel et al. 2021 [23] | To explore participants' user experience of the telehealth visit using the CPATH. | QUAL: qualitative description | smartphone technology | a remote APRN | 9 patients and caregivers | A large hospice agency | NA | the usability of CPATH, the perspectives of patients' caregivers | Participants prefer a blended model of in-person and telehealth visits, with telehealth suitable for routine visits. In-person assessments are needed if remote communication is inadequate. |
| Iran | Alizadeh et al. 2023 [24] | To explore the current barriers of integration of palliative care services from hospital to home for cancer patients during the COVID-19 Pandemic. | QUAL: qualitative description | NA | NA | NA | NA | NA | current barriers of integration of PC services from hospital to home for cancer patients during the COVID-19 | Four main challenges in HBPC identified: education, implementation, policy, and drug availability. |
| Swizerland | Ebneter et al. 2024 [25] | To identify the perceptions and needs of healthcare professionals regarding telemedicine in a Swiss outpatient PC network. | MMR: convergent parallel design | digital care conference | nurses (home care, home care with training in PC, SPC) and physicians (primary care, specialist, PC) | NA | local palliative care service/network Mobile Palliative Care Service Bern Area (rural and urban) | NA | the perceptions and needs of healthcare professionals | Telemedicine was seen as beneficial for easy access to HCPs and better communication while it faced barriers such as lack of acceptance, physical contact, data breach concerns, and technical issues. |
| The Gold Line, UK | Middleton-Green et al. 2016 [26] | To evaluate the support service for patients. | MMR: convergent parallel design | 24/7 telephone and video consultation | a nursing team | 4648 patients for survey; 5 patients for interviews and 7 carers for interviews | community and Airedale general hospital | 24/7 services | Activity and other data recorded at the time of calls were analyzed; patients and carers' perspectives. | From April 2014 to March 2015, the Gold Line registered 4648 patients. The service handled 4533 calls and 573 video consultations, with 39% resolved without referrals. |
| Oncokompas, the Netherlands | de Veer et al. 2020 [27] | To gain an understanding of the perceptions of patients with incurable cancer. | MMR: explanatory sequential design | an application (Oncokompas) | homecare nurses | 36 patients with cancer | 4 homecare organiazations | NA | Patient: experiences, activation (PAM), QOL (EORTC QLQ-C15-PAL); satisfaction | 85% of patients were satisfied with the assessment of their needs and the advice received. They valued nurses’ expertise and the assistance provided in their homes. |
| HomePal, USA | Osuji et al. 2020 [28] and Nguyen et al. 2020 [29] | To compare end-of life care in decedents who received HomePal with two cohorts, assess clinician experience with video visit implementation, and describe the challenges in conducting the HomePal study. | MMR: explanatory sequential design | video visits | registered homecare nurses as case manager and a remote physician | 3533 patients and 463 caregivers | 14 sites of a large integrated healthcare system in Kaiser Permanente Southern California | NA | Patient: symptom burden (ESAS), general distress and QOL (PROMIS-10); caregiving preparedness (PCS, PROMIS-10, ZBI-12); nurses’ experiences; medical resource use: days at home in the last 6 months | Interviews highlighted two themes: positive patient and family responses and convenience enhancing value, while technology issues and scheduling challenges diminished it. |
| USA | Cameron, 2021 [30] | To examine the comfort of hospice staff using telehospice to connect virtually with patients and caregivers. | MMR: convergent parallel design | a remote patient monitoring and communication platform | hospice nurses, NPs, physicians, etc. | NA | a hospice | 24/7 services | comfort level, experiences and perspectives of using AVA | Three themes emerged from the qualitative responses: Positive responses about AVA; AVA as an assistive device; and Problems with connectivity. |
| EPIC, USA | Iyer et al. 2023 [31] | To conduct a formative and summative evaluation of EPIC. | MMR: multi-phases design | telephonic coaching | 2 registered PC nurse coaches | phase 1: 10 patients, 10 family caregivers; phase 2: 5 patients; phase, 5 family caregivers | pulmonary clinics | NA | intervention and data collection feasibility (≥70% completion) | EPIC were acceptable and feasible to support adaptation. |
| RELIEF, Canada | Bhargava et al. 2021 [32] | To demonstrate RELIEF to be a feasible tool for patients. | MMR: explanatory sequential design | APP | primary PC nurses and phisicians | 13 patients | a large community hospital | monitoring RELIEF alerts daily | use and usability of RELIEF  emergency department visits | RELIEF is a feasible and acceptable method for the remote monitoring of patients with palliative care needs through regular symptom self-reporting. |
| Australia | Jiang et al. 2023 [33] | To assess the feasibility of integrating TH-SPC into a rural community setting. | MMR: convergent parallel design | video consultation | community PC team and metropolitan-located SPC team (nurses and physicians) | 21 patients (14 to the TH-SPC intervention and 7 to standard care) | a rural community setting | as needed | Feasibility outcomes included efficiency of process, user satisfaction, clinical outcome and health-care metrics. | TH-SPC was successfully integrated into rural community-based palliative care, with potential benefits in performance status preservation and health-care resource utilisation. |
| Canada | Read Paul et al. 2019 [34] | To gain an understanding of the experience of using mobile WBVC for conducting in home palliative care consults. | MMR: convergent parallel design | in-person homecare and video consultation | homecare nurses and a rural PC consultation team (PC CNSs and physicians) | 10 patients and 13 family caregivers | a rural area | NA | Experience and audiovisual quality | Using WBVC for in-home palliative care consults could be an acceptable, effective, feasible, and efficient way to provide timely support to elderly rural patients and their families. |

References

1. Bakitas MA, Tosteson TD, Li Z, et al. Early versus delayed initiation of concurrent palliative oncology care: patient outcomes in the ENABLE III randomized controlled trial. J Clin Oncol 2015;33(13):1438-1445. doi:10.1200/JCO.2014.58.6362
2. Dionne-Odom JN, Azuero A, Lyons KD, et al. Benefits of early versus delayed palliative care to informal family caregivers of patients with advanced cancer: outcomes from the ENABLE III randomized controlled trial. J Clin Oncol 2015;33(13):1446-1452. doi:10.1200/JCO.2014.58.7824
3. Bakitas MA, Dionne-Odom JN, Ejem DB, et al. Effect of an early palliative care telehealth intervention vs usual care on patients with heart failure: the ENABLE CHF-PC randomized clinical trial. JAMA Intern Med 2020;180(9):1203-1213. doi:10.1001/jamainternmed.2020.2861
4. Dionne-Odom JN, Ejem DB, Wells R, et al. Effects of a telehealth early palliative care intervention for family caregivers of persons with advanced heart failure: the ENABLE CHF-PC randomized clinical trial. JAMA Netw Open 2020;3(4):e202583. doi:10.1001/jamanetworkopen.2020.2583
5. Wilkie DJ, Yao Y, Ezenwa MO, et al. A stepped-wedge randomized controlled trial: effects of eHealth interventions for pain control among adults with cancer in hospice. J Pain Symptom Manage 2020;59(3):626-636. doi:10.1016/j.jpainsymman.2019.10.028
6. Schoppee TM, Dyal BW, Scarton L, et al. Patients and caregivers rate the PAINReportIt wireless internet-enabled tablet as a method for reporting pain during end-of-life cancer care. Cancer Nurs 2020;43(5):419-424. doi:10.1097/NCC.0000000000000743
7. Bekelman DB, Feser W, Morgan B, et al. Nurse and Social Worker Palliative Telecare Team and Quality of Life in Patients With COPD, Heart Failure, or Interstitial Lung Disease: The ADAPT Randomized Clinical Trial [published correction appears in JAMA. 2025 Mar 26. doi: 10.1001/jama.2025.3767.]. JAMA. 2024;331(3):212-223. doi:10.1001/jama.2023.24035
8. Hoek PD, Schers HJ, Bronkhorst EM, et al. The effect of weekly specialist palliative care teleconsultations in patients with advanced cancer—a randomized clinical trial. BMC Med 2017;15(1):119. doi:10.1186/s12916-017-0866-9
9. Doorenbos AZ, Levy WC, Curtis JR, et al. An intervention to enhance goals-of-care communication between heart failure patients and heart failure providers. J Pain Symptom Manage 2016;52(3):353-360. doi:10.1016/j.jpainsymman.2016.03.018
10. Mirshahi A, Bakitas M, Khoshavi M, et al. The impact of an integrated early palliative care telehealth intervention on the quality of life of heart failure patients: a randomized controlled feasibility study. BMC Palliat Care 2024;23(1):22. doi:10.1186/s12904-024-01348-z
11. Schmucker AM, Flannery M, Cho J, et al. Data from emergency medicine palliative care access (EMPallA): a randomized controlled trial comparing the effectiveness of specialty outpatient versus telephonic palliative care of older adults with advanced illness presenting to the emergency department. BMC Emerg Med 2021;21(1):83. doi:10.1186/s12873-021-00478-4
12. Vitacca M, Comini L, Tabaglio E, et al. Tele-assisted palliative homecare for advanced chronic obstructive pulmonary disease: a feasibility study. J Palliat Med 2019;22(2):173-178. doi:10.1089/jpm.2018.0321
13. Dionne-Odom JN, Williams GR, Warren PP, et al. Implementing a clinic-based telehealth support service (FamilyStrong) for family caregivers of individuals with grade IV brain tumors. J Palliat Med 2021;24(3):347-353. doi:10.1089/jpm.2020.0178
14. Valenti V, Rossi R, Scarpi E, et al. Nurse-led telephone follow-up for early palliative care patients with advanced cancer. J Clin Nurs 2023;32(11-12):2846-2853. doi:10.1111/jocn.16403
15. Balasubramanian S, Biji MS, Ranjith MK, et al. Patient satisfaction in home care services through e-palliative care—an experience of tertiary cancer centre from Kerala. Indian J Palliat Care 2022;28(3):250-255. doi:10.25259/IJPC_36_2021
16. Evering RMH, Postel MG, van Os-Medendorp H, et al. Intention of healthcare providers to use video-communication in terminal care: a cross-sectional study. BMC Palliat Care 2022;21(1):213. doi:10.1186/s12904-022-01100-5
17. Guo J, Dai Y, Gong Y, et al. Exploring the telehealth readiness and its related factors among palliative care specialist nurses: a cross-sectional study in China. BMC Palliat Care 2023;22(1):82. doi:10.1186/s12904-023-01209-1
18. Salem R, El Zakhem A, Gharamti A, et al. Palliative care via telemedicine: a qualitative study of caregiver and provider perceptions. J Palliat Med 2020;23(12):1594-1598. doi:10.1089/jpm.2020.0002
19. Funderskov KF, Boe Danbjørg D, Jess M, Munk L, Olsen Zwisler AD, Dieperink KB. Telemedicine in specialised palliative care: Healthcare professionals' and their perspectives on video consultations-A qualitative study. J Clin Nurs. 2019;28(21-22):3966-3976. doi:10.1111/jocn.15004
20. Funderskov KF, Raunkiær M, Danbjørg DB, et al. Experiences With Video Consultations in Specialized Palliative Home-Care: Qualitative Study of Patient and Relative Perspectives. J Med Internet Res. 2019;21(3):e10208. Published 2019 Mar 21. doi:10.2196/10208
21. Vincent D, Peixoto C, Quinn KL, et al. Virtual home-based palliative care during COVID-19: a qualitative exploration of the patient, caregiver, and healthcare provider experience. Palliat Med 2022;36(9):1374-1388. doi:10.1177/02692163221116251
22. Oelschlägel L, Dihle A, Christensen VL, et al. Implementing welfare technology in palliative homecare for patients with cancer: a qualitative study of health-care professionals’ experiences. BMC Palliat Care 2021;20(1):146. doi:10.1186/s12904-021-00844-w
23. Bethel C, Towers V, Crist JD, et al. A guide for intentional home telehealth assessment: patient and caregiver perceptions. CIN Comput Inform Nurs 2021;39(12):943-947. doi:10.1097/CIN.0000000000000779
24. Alizadeh Z, Rohani C, Rassouli M, et al. Challenges of integrated home-based palliative care services for cancer patients during the COVID-19 pandemic: a qualitative content analysis. Home Health Care Manag Pract 2023;35(3):180-189. doi:10.1177/10848223221134780
25. Ebnester AS, Maessen M, Sauter TC, et al. Perceptions and needs of an outpatient palliative care team regarding digital care conferences in palliative care: a mixed-method online survey. Swiss Med Wkly 2024;154(1):3487. doi:10.57187/s.3487
26. Middleton-Green L, Gadoud A, Norris B, et al. “A friend in the corner”: supporting people at home in the last year of life via telephone and video consultation—an evaluation. BMJ Support Palliat Care 2016;9(4):e26. doi:10.1136/bmjspcare-2015-001016
27. De Veer AJE, Slev VN, Pasman HR, et al. Assessment of a structured self-management support intervention by nurses for patients with incurable cancer. Oncol Nurs Forum 2020;47(3):305-317. doi:10.1188/20.ONF.305-317
28. Osuji TA, Macias M, McMullen C, et al. Clinician perspectives on implementing video visits in home-based palliative care. Palliat Med Rep 2020;1(1):221-226. doi:10.1089/pmr.2020.0074
29. Nguyen HQ, McMullen C, Haupt EC, et al. Findings and lessons learnt from early termination of a pragmatic comparative effectiveness trial of video consultations in home-based palliative care. BMJ Support Palliat Care. Published online October 13, 2020. doi:10.1136/bmjspcare-2020-002553
30. Cameron P. Hospice staff comfort with telehospice. Home Healthc Now 2021;39(6):344-350. doi:10.1097/NHH.0000000000001019
31. Iyer AS, Wells RD, Dionne-Odom JN, et al. Project EPIC (Early Palliative Care in COPD): a formative and summative evaluation of the EPIC telehealth intervention. J Pain Symptom Manage 2023;65(4):335-347.e3. doi:10.1016/j.jpainsymman.2022.11.024
32. Bhargava R, Keating B, Isenberg SR, et al. RELIEF: a digital health tool for the remote self-reporting of symptoms in patients with cancer to address palliative care needs and minimize emergency department visits. Curr Oncol 2021;28(6):4273-4280. doi:10.3390/curroncol28060363
33. Jiang B, Bills M, Poon P. Integrated telehealth-assisted home-based specialist palliative care in rural Australia: a feasibility study. J Telemed Telecare 2023;29(1):50-57. doi:10.1177/1357633X20966466
34. Read Paul L, Salmon C, Sinnarajah A, Spice R. Web-based videoconferencing for rural palliative care consultation with elderly patients at home. Support Care Cancer. 2019;27(9):3321-3330. doi:10.1007/s00520-018-4580-8
