# Supplementary material for: Nurse-Delivered Telehealth in Home-Based Palliative Care: Integrative Systematic Review
Source: J Med Internet Res. 2025 May 5;27:e73024. doi: 10.2196/73024 (PMC12089877; doi:10.2196/73024)
Supplement: Multimedia Appendix 4 [file jmir_v27i1e73024_app4.docx]

**Mixed Methods Appraisal Tool (MMAT), version 2018**

1. **Category of study designs: Qualitative**

| **Author** | **Methodological quality criteria** | | | | | | | **comments** |
| --- | --- | --- | --- | --- | --- | --- | --- | --- |
|  | S1. Are there clear research questions? | S2. Do the collected data allow to address the research questions? | 1.1. Is the qualitative approach appropriate to answer the research question? | 1.2. Are the qualitative data collection methods adequate to address the research question? | 1.3. Are the findings adequately derived from the data? | 1.4. Is the interpretation of results sufficiently substantiated by data? | 1.5. Is there coherence between qualitative data sources, collection, analysis and interpretation? |  |
| Salem et al. 2020^[1]^ | Yes | Yes | Yes | Can’t tell | Yes | Yes | Yes | Insufficient details on interview guide design. |
| Funderskov et al. 2019 (1)^[2]^ | Yes | Yes | Yes | Yes | Yes | Yes | Yes |  |
| Funderskov et al. 2019 (2)^[3]^ | Yes | Yes | Yes | Yes | Yes | Yes | Yes |  |
| Vincent et al. 2022^[4]^ | Yes | Yes | Yes | Yes | Yes | Yes | Yes |  |
| Oelschlägel et al. 2021^[5]^ | Yes | Yes | Yes | Can’t tell | Yes | Yes | Can’t tell | Insufficient description of how focus groups and individual interviews were triangulated. |
| Bethel et al. 2021^[6]^ | Yes | Yes | Yes | Yes | Yes | Yes | Can’t tell | Some themes (e.g., privacy concerns) lacked direct quotes to substantiate interpretations. |
| Alizadeh et al. 2023 ^[7]^ | Yes | Yes | Yes | Yes | Yes | Yes | Yes |  |

1. **Category of study designs: Quantitative randomized controlled trials**

| **Author** | **Methodological quality criteria** | | | | | | | **comments** |
| --- | --- | --- | --- | --- | --- | --- | --- | --- |
|  | S1. Are there clear research questions? | S2. Do the collected data allow to address the research questions? | 2.1. Is randomization appropriately performed? | 2.2. Are the groups comparable at baseline? | 2.3. Are there complete outcome data? | 2.4. Are outcome assessors blinded to the intervention provided? | 2.5. Did the participants adhere to the assigned intervention? |  |
| Bakitas et al. 2015^[8]^ | Yes | Yes | Yes | No | Can’t tell | No | No | Missing data handling (e.g., attrition rate) not explicitly described.  Outcome assessors were blinded, but participants/providers were not, risking performance bias. |
| Dionne-Odom et al. 2015^[9]^ | Yes | Yes | Can’t tell | Yes | Yes | Can’t tell | Can’t tell | Randomization and stratification are mentioned, but the method is not detailed. Blinding of outcome assessors is not described and adherence data (e.g., completion rate) are missing. |
| Bakitas et al. 2020^[10]^ | Yes | Yes | Yes | Yes | Yes | Can’t tell | No | Blinding of outcome assessors is unclear. Low adherence. |
| Dionne-Odom et al. 2020^[11]^ | Yes | Yes | Yes | Yes | No | No | No | Participants and intervention providers were not blinded. Low adherence. |
| Wilkie et al. 2020^[12]^ | Yes | Yes | Can’t tell | Yes | Yes | No | No | No details on sequence generation or allocation concealment. Nurses and data collectors were aware of group assignment. Low adherence. |
| Bekelman et al. 2024^[13]^ | Yes | Yes | Yes | Yes | Yes | No | Yes | Single-blind design (participants unblinded), but no mention of blinding outcome assessors. Risk of detection bias. |
| Hoek et al. 2017^[14]^ | Yes | Yes | Yes | Yes | No | No | Yes | High attrition rates and lack of explicit missing data handling. The study was non-blinded. |
| Doorenbos et al. 2016^[15]^ | Yes | Yes | Can’t tell | Yes | Yes | No | Can’t tell | Lacks details on randomization methods. Blinding of outcome assessors is unclear. No data on intervention adherence. |
| Mirshahi et al. 2024 ^[16]^ | Yes | Yes | Can’t tell | Yes | Yes | Yes | Can’t tell | Allocation hiding measures are not explained, and there is selection bias |
| Schmucker et al. 2021 ^[17]^ | Yes | Yes | Can’t tell | Yes | Can’t tell | Yes | Yes | Insufficient details on randomization/allocation concealment, and incomplete reporting of attrition. |
| Nguyen et al. 2020^[18]^ | Yes | Yes | Can’t tell | Yes | No | No | Yes | The randomization method was not explicitly described. The study was terminated early. |

1. **Category of study designs: Quantitative non-randomized**

| **Author** | **Methodological quality criteria** | | | | | | | **comments** |
| --- | --- | --- | --- | --- | --- | --- | --- | --- |
|  | S1. Are there clear research questions? | S2. Do the collected data allow to address the research questions? | 3.1. Are the participants representative of the target population? | 3.2. Are measurements appropriate regarding both the outcome and intervention (or exposure)? | 3.3. Are there complete outcome data? | 3.4. Are the confounders accounted for in the design and analysis? | 3.5. During the study period, is the intervention administered (or exposure occurred) as intended? |  |
| Vitacca et al. 2019 ^[19]^ | Yes | Yes | Can’t tell | Yes | Yes | No | Can’t tell | No adjustment for confounders in analysis. |
| Dionne-Odom et al. 2021^[20]^ | Yes | Yes | Yes | Yes | Yes | No | Can’t tell | No adjustment for confounders in analysis. No fidelity data were reported. |
| Valenti et al. 2023^[21]^ | Yes | Yes | Yes | Yes | Can’t tell | No | Can’t tell | No information on missing data was provided. No adjustment for confounders in analysis. No fidelity data were reported. |

1. **Category of study designs: Quantitative descriptive**

| **Author** | **Methodological quality criteria** | | | | | | | **comments** |
| --- | --- | --- | --- | --- | --- | --- | --- | --- |
|  | S1. Are there clear research questions? | S2. Do the collected data allow to address the research questions? | 4.1. Is the sampling strategy relevant to address the research question? | 4.2. Is the sample representative of the target population? | 4.3. Are the measurements appropriate? | 4.4. Is the risk of nonresponse bias low? | 4.5. Is the statistical analysis appropriate to answer the research question? |  |
| Schoppee et al. 2020^[22]^ | Yes | Yes | Can’t tell | Yes | Can’t tell | Can’t tell | Yes | The sampling method and response rate was not reported. |
| Balasubramanian et al. 2022^[23]^ | Yes | Yes | Yes | Yes | Can’t tell | Yes | Yes | The measurement tool did not specify the specific reliability and validity, and the research results were incomplete. |
| Evering et al. 2022 ^[24]^ | Yes | Yes | Yes | Yes | No | Yes | Yes | Low internal consistency in some constructs (e.g., α=0.446) reduces validity. |
| Guo et al. 2023 ^[25]^ | Yes | Yes | Yes | Yes | Yes | Yes | Yes |  |

1. **Category of study designs: Mixed methods**

| **Author** | **Methodological quality criteria** | | | | | | | **comments** |
| --- | --- | --- | --- | --- | --- | --- | --- | --- |
|  | S1. Are there clear research questions? | S2. Do the collected data allow to address the research questions? | 5.1. Is there an adequate rationale for using a mixed methods design to address the research question? | 5.2. Are the different components of the study effectively integrated to answer the research question? | 5.3. Are the outputs of the integration of qualitative and quantitative components adequately interpreted? | 5.4. Are divergences and inconsistencies between quantitative and qualitative results adequately addressed? | 5.5. Do the different components of the study adhere to the quality criteria of each tradition of the methods involved? |  |
| Ebneter et al. 2024^[26]^ | Yes | Yes | Yes | Can’t tell | No | Yes | Yes | Integration strategies are not explicitly described. Results are presented separately. |
| Middleton-Green et al. 2016^[27]^ | Yes | Yes | Yes | Can’t tell | No | Yes | Yes | Integration strategies are not explicitly described. Results are presented separately. |
| de Veer et al. 2020^[28]^ | Yes | Yes | Yes | Can’t tell | No | Yes | Yes | Integration strategies are not explicitly described. Results are presented separately. |
| Osuji et al. 2020^[29]^ | Yes | Yes | Yes | Can’t tell | No | Yes | Yes | Integration strategies are not explicitly described. Results are presented separately. |
| Cameron 2021^[30]^ | Yes | Yes | Yes | Yes | No | No | No | The survey's response rate was low, and the reasons were not analyzed. |
| Iyer et al. 2023 ^[31]^ | Yes | Yes | Yes | Yes | Can’t tell | No | Can’t tell | The article does not address potential discrepancies between qualitative and quantitative findings. |
| Bhargava et al. 2021 ^[32]^ | Yes | Yes | Yes | Can’t tell | No | Yes | Can’t tell | Integration strategies are not explicitly described. Results are presented separately. |
| Jiang et al. 2023 ^[33]^ | Yes | Yes | Yes | No | Can’t tell | Yes | Can’t tell | No details on data collection/analysis and insufficient information on controlling confounders. |
| Read Paul et al. 2019 ^[34]^ | Yes | Yes | Yes | No | Can’t tell | Yes | Can’t tell | No evidence of integration. |

References

[1] SALEM R, EL ZAKHEM A, GHARAMTI A, 等. Palliative Care via Telemedicine: A Qualitative Study of Caregiver and Provider Perceptions[J/OL]. Journal of Palliative Medicine, 2020, 23(12): 1594-1598. DOI:10.1089/jpm.2020.0002.

[2] FUNDERSKOV K F, BOE DANBJØRG D, JESS M, 等. Telemedicine in specialised palliative care: Healthcare professionals’ and their perspectives on video consultations—A qualitative study[J/OL]. Journal of Clinical Nursing, 2019, 28(21-22): 3966-3976. DOI:10.1111/jocn.15004.

[3] FUNDERSKOV K F, RAUNKIÆR M, DANBJØRG D B, 等. Experiences With Video Consultations in Specialized Palliative Home-Care: Qualitative Study of Patient and Relative Perspectives[J/OL]. Journal of Medical Internet Research, 2019, 21(3): e10208. DOI:10.2196/10208.

[4] VINCENT D, PEIXOTO C, QUINN K L, 等. Virtual home-based palliative care during COVID-19: A qualitative exploration of the patient, caregiver, and healthcare provider experience[J/OL]. Palliative Medicine, 2022, 36(9): 1374-1388. DOI:10.1177/02692163221116251.

[5] OELSCHLÄGEL L, DIHLE A, CHRISTENSEN V L, 等. Implementing welfare technology in palliative homecare for patients with cancer: a qualitative study of health-care professionals’ experiences[J/OL]. BMC palliative care, 2021, 20(1): 146. DOI:10.1186/s12904-021-00844-w.

[6] BETHEL C, TOWERS V, CRIST J D, 等. A Guide for Intentional Home Telehealth Assessment: Patient and Caregiver Perceptions[J/OL]. CIN: Computers, Informatics, Nursing, 2021, 39(12): 943-947. DOI:10.1097/CIN.0000000000000779.

[7] ALIZADEH Z, ROHANI C, RASSOULI M, 等. Challenges of Integrated Home-Based Palliative Care Services for Cancer Patients during the COVID-19 Pandemic: A Qualitative Content Analysis[J/OL]. Home Health Care Management & Practice, 2023, 35(3): 180-189. DOI:10.1177/10848223221134780.

[8] BAKITAS M A, TOSTESON T D, LI Z, 等. Early Versus Delayed Initiation of Concurrent Palliative Oncology Care: Patient Outcomes in the ENABLE III Randomized Controlled Trial[J/OL]. Journal of Clinical Oncology: Official Journal of the American Society of Clinical Oncology, 2015, 33(13): 1438-1445. DOI:10.1200/JCO.2014.58.6362.

[9] DIONNE-ODOM J N, AZUERO A, LYONS K D, 等. Benefits of Early Versus Delayed Palliative Care to Informal Family Caregivers of Patients With Advanced Cancer: Outcomes From the ENABLE III Randomized Controlled Trial[J/OL]. Journal of Clinical Oncology: Official Journal of the American Society of Clinical Oncology, 2015, 33(13): 1446-1452. DOI:10.1200/JCO.2014.58.7824.

[10] BAKITAS M A, DIONNE-ODOM J N, EJEM D B, 等. Effect of an Early Palliative Care Telehealth Intervention vs Usual Care on Patients With Heart Failure: The ENABLE CHF-PC Randomized Clinical Trial[J/OL]. JAMA internal medicine, 2020, 180(9): 1203-1213. DOI:10.1001/jamainternmed.2020.2861.

[11] DIONNE-ODOM J N, EJEM D B, WELLS R, 等. Effects of a Telehealth Early Palliative Care Intervention for Family Caregivers of Persons With Advanced Heart Failure: The ENABLE CHF-PC Randomized Clinical Trial[J/OL]. JAMA network open, 2020, 3(4): e202583. DOI:10.1001/jamanetworkopen.2020.2583.

[12] WILKIE D J, YAO Y, EZENWA M O, 等. A Stepped-Wedge Randomized Controlled Trial: Effects of eHealth Interventions for Pain Control Among Adults With Cancer in Hospice[J/OL]. Journal of Pain and Symptom Management, 2020, 59(3): 626-636. DOI:10.1016/j.jpainsymman.2019.10.028.

[13] BEKELMAN D B, FESER W, MORGAN B, 等. Nurse and Social Worker Palliative Telecare Team and Quality of Life in Patients With COPD, Heart Failure, or Interstitial Lung Disease[J]. JAMA, 2024, 331(3): 212-223.

[14] HOEK P D, SCHERS H J, BRONKHORST E M, 等. The effect of weekly specialist palliative care teleconsultations in patients with advanced cancer –a randomized clinical trial[J/OL]. BMC Medicine, 2017, 15(1): 119. DOI:10.1186/s12916-017-0866-9.

[15] DOORENBOS A Z, LEVY W C, CURTIS J R, 等. An Intervention to Enhance Goals-of-Care Communication Between Heart Failure Patients and Heart Failure Providers[J/OL]. Journal of Pain and Symptom Management, 2016, 52(3): 353-360. DOI:10.1016/j.jpainsymman.2016.03.018.

[16] MIRSHAHI A, BAKITAS M, KHOSHAVI M, 等. The impact of an integrated early palliative care telehealth intervention on the quality of life of heart failure patients: a randomized controlled feasibility study[J/OL]. BMC palliative care, 2024, 23(1): 22. DOI:10.1186/s12904-024-01348-z.

[17] SCHMUCKER A M, FLANNERY M, CHO J, 等. Data from emergency medicine palliative care access (EMPallA): a randomized controlled trial comparing the effectiveness of specialty outpatient versus telephonic palliative care of older adults with advanced illness presenting to the emergency department[J/OL]. BMC emergency medicine, 2021, 21(1): 83. DOI:10.1186/s12873-021-00478-4.

[18] NGUYEN H Q, MCMULLEN C, HAUPT E C, 等. Findings and lessons learnt from early termination of a pragmatic comparative effectiveness trial of video consultations in home-based palliative care[J/OL]. BMJ supportive & palliative care, 2020: bmjspcare-2020-002553. DOI:10.1136/bmjspcare-2020-002553.

[19] VITACCA M, COMINI L, TABAGLIO E, 等. Tele-Assisted Palliative Homecare for Advanced Chronic Obstructive Pulmonary Disease: A Feasibility Study[J/OL]. Journal of Palliative Medicine, 2019, 22(2): 173-178. DOI:10.1089/jpm.2018.0321.

[20] DIONNE-ODOM J N, WILLIAMS G R, WARREN P P, 等. Implementing a Clinic-Based Telehealth Support Service (FamilyStrong) for Family Caregivers of Individuals with Grade IV Brain Tumors[J/OL]. Journal of Palliative Medicine, 2021, 24(3): 347-353. DOI:10.1089/jpm.2020.0178.

[21] VALENTI V, ROSSI R, SCARPI E, 等. Nurse-led telephone follow-up for early palliative care patients with advanced cancer[J/OL]. Journal of Clinical Nursing, 2023, 32(11-12): 2846-2853. DOI:10.1111/jocn.16403.

[22] SCHOPPEE T M, DYAL B W, SCARTON L, 等. Patients and Caregivers Rate the PAINReportIt Wireless Internet-Enabled Tablet as a Method for Reporting Pain During End-of-Life Cancer Care[J/OL]. Cancer Nursing, 2020, 43(5): 419-424. DOI:10.1097/NCC.0000000000000743.

[23] BALASUBRAMANIAN S, BIJI M S, RANJITH M K, 等. Patient satisfaction in Home care services through e-Palliative Care -An experience of tertiary cancer centre from Kerala[J/OL]. Indian Journal of Palliative Care, 2022, 28(3): 250-255. DOI:10.25259/IJPC_36_2021.

[24] EVERING R M H, POSTEL M G, VAN OS-MEDENDORP H, 等. Intention of healthcare providers to use video-communication in terminal care: a cross-sectional study[J/OL]. BMC palliative care, 2022, 21(1): 213. DOI:10.1186/s12904-022-01100-5.

[25] GUO J, DAI Y, GONG Y, 等. Exploring the telehealth readiness and its related factors among palliative care specialist nurses: a cross-sectional study in China[J/OL]. BMC palliative care, 2023, 22(1): 82. DOI:10.1186/s12904-023-01209-1.

[26] EBNETER A S, MAESSEN M, SAUTER T C, 等. Perceptions and needs of an outpatient palliative care team regarding digital care conferences in palliative care: a mixed-method online survey[J/OL]. Swiss Medical Weekly, 2024, 154(1): 3487. DOI:10.57187/s.3487.

[27] MIDDLETON-GREEN L, GADOUD A, NORRIS B, 等. “A Friend in the Corner”: supporting people at home in the last year of life via telephone and video consultation-an evaluation[J/OL]. BMJ supportive & palliative care, 2016, 9(4): e26. DOI:10.1136/bmjspcare-2015-001016.

[28] DE VEER A J E, SLEV V N, PASMAN H R, 等. Assessment of a Structured Self-Management Support Intervention by Nurses for Patients With Incurable Cancer[J/OL]. Oncology Nursing Forum, 2020, 47(3): 305-317. DOI:10.1188/20.ONF.305-317.

[29] OSUJI T A, MACIAS M, MCMULLEN C, 等. Clinician Perspectives on Implementing Video Visits in Home-Based Palliative Care[J/OL]. Palliative Medicine Reports, 2020, 1(1): 221-226. DOI:10.1089/pmr.2020.0074.

[30] CAMERON P. Hospice Staff Comfort with Telehospice[J/OL]. Home Healthcare Now, 2021, 39(6): 344-350. DOI:10.1097/NHH.0000000000001019.

[31] IYER A S, WELLS R D, DIONNE-ODOM J N, 等. Project EPIC (Early Palliative Care In COPD): A Formative and Summative Evaluation of the EPIC Telehealth Intervention[J/OL]. Journal of Pain and Symptom Management, 2023, 65(4): 335-347.e3. DOI:10.1016/j.jpainsymman.2022.11.024.

[32] BHARGAVA R, KEATING B, ISENBERG S R, 等. RELIEF: A Digital Health Tool for the Remote Self-Reporting of Symptoms in Patients with Cancer to Address Palliative Care Needs and Minimize Emergency Department Visits[J/OL]. Current Oncology (Toronto, Ont.), 2021, 28(6): 4273-4280. DOI:10.3390/curroncol28060363.

[33] JIANG B, BILLS M, POON P. Integrated telehealth-assisted home-based specialist palliative care in rural Australia: A feasibility study[J/OL]. Journal of Telemedicine and Telecare, 2023, 29(1): 50-57. DOI:10.1177/1357633X20966466.

[34] READ PAUL L, SALMON C, SINNARAJAH A, 等. Web-based videoconferencing for rural palliative care consultation with elderly patients at home[J/OL]. Supportive Care in Cancer, 2019, 27(9): 3321-3330. DOI:10.1007/s00520-018-4580-8.
